# Structural features and development of an assay platform of the parasite target deoxyhypusine synthase of *Brugia malayi* and *Leishmania major*

**DOI:** 10.1371/journal.pntd.0008762

**Published:** 2020-10-12

**Authors:** Suélen Fernandes Silva, Angélica Hollunder Klippel, Priscila Zonzini Ramos, André da Silva Santiago, Sandro Roberto Valentini, Mario Henrique Bengtson, Katlin Brauer Massirer, Elizabeth Bilsland, Rafael Miguez Couñago, Cleslei Fernando Zanelli

**Affiliations:** 1 Chemistry Institute, São Paulo State University—UNESP, Araraquara, SP, Brazil; 2 School of Pharmaceutical Sciences, São Paulo State University—UNESP, Araraquara, SP, Brazil; 3 Molecular Biology and Genetic Engineering Center (CBMEG), Medicinal Chemistry Center (CQMED), Structural Genomics Consortium (SGC-UNICAMP), University of Campinas-UNICAMP, Campinas, SP, Brazil; 4 Department of Structural and Functional Biology, Institute of Biology, University of Campinas—UNICAMP, Campinas, SP, Brazil; 5 Department of Biochemistry and Tissue Biology, Institute of Biology, University of Campinas—UNICAMP, Campinas, SP, Brazil; University of Liverpool, UNITED KINGDOM

## Abstract

Deoxyhypusine synthase (DHS) catalyzes the first step of the post-translational modification of eukaryotic translation factor 5A (eIF5A), which is the only known protein containing the amino acid hypusine. Both proteins are essential for eukaryotic cell viability, and DHS has been suggested as a good candidate target for small molecule-based therapies against eukaryotic pathogens. In this work, we focused on the DHS enzymes from *Brugia malayi* and *Leishmania major*, the causative agents of lymphatic filariasis and cutaneous leishmaniasis, respectively. To enable *B*. *malayi* (Bm)DHS for future target-based drug discovery programs, we determined its crystal structure bound to cofactor NAD^+^. We also reported an *in vitro* biochemical assay for this enzyme that is amenable to a high-throughput screening format. The *L*. *major* genome encodes two DHS paralogs, and attempts to produce them recombinantly in bacterial cells were not successful. Nevertheless, we showed that ectopic expression of both LmDHS paralogs can rescue yeast cells lacking the endogenous DHS-encoding gene (*dys1*). Thus, functionally complemented *dys1Δ* yeast mutants can be used to screen for new inhibitors of the *L*. *major* enzyme. We used the known human DHS inhibitor GC7 to validate both *in vitro* and yeast-based DHS assays. Our results show that BmDHS is a homotetrameric enzyme that shares many features with its human homologue, whereas LmDHS paralogs are likely to form a heterotetrameric complex and have a distinct regulatory mechanism. We expect our work to facilitate the identification and development of new DHS inhibitors that can be used to validate these enzymes as vulnerable targets for therapeutic interventions against *B*. *malayi* and *L*. *major* infections.

## Author summary

Target-based drug discovery strategies hold the promise to discover safer and more effective treatments for Neglected Tropical Diseases (NTDs). Genetic manipulation techniques have been used to successfully identify essential genes in eukaryotic parasites. Unfortunately, the fact that a gene is essential under controlled laboratory conditions does not automatically make the corresponding gene-product vulnerable to pharmacological intervention in a clinical setting within the human host. To allow the discovery and development of small molecule tool compounds that can be used to validate pharmacologically vulnerable targets, one must first establish compound screening assays and obtain structural information for the candidate target. Eukaryotic cells lacking deoxyhypusine synthase (DHS) function are not viable. DHS catalyzes the first step in a post-translational modification that is critical for the function of eIF5A. Presence of mature eIF5A is also essential for eukaryotic cell viability. Here we reported compound screening assays (yeast-based for *Brugia malayi* and *Leishmania major*; *in vitro* for *B*. *malayi* only) and provided further regulatory and structural insights we hope will aid in the identification and development of inhibitors for the DHS enzymes from two NTD-causing organisms—*B*. *malayi*, the causative agent of lymphatic filariasis and *L*. *major*, the causative agent of cutaneous leishmaniasis.

## Introduction

Neglected tropical diseases (NTDs) affect over 1.5 billion people and cause approximately 500,000 deaths per year, mainly in low- and middle-income countries [1]. Two NTDs in urgent need of new treatments are leishmaniasis, caused by *Leishmania* parasites, and lymphatic filariasis, caused by tissue-dwelling nematodes *Wuchereria bancrofti*, *Brugia malayi* and *Brugia timori*. Current treatments for the three main clinical manifestations of leishmaniasis (cutaneous, mucocutaneous and visceral) have severe side-effects and many are currently ineffective due to the emergence of resistance [2,3]. For lymphatic filariasis, eradication efforts have been hampered by the inability of current drugs to kill adult worms [4,5]. Currently, there are approximately 70 million people infected with filarial worms and up to 1.6 million new leishmaniasis cases each year [1,6].

Target-based drug discovery strategies hold the promise to help develop safer and more effective medicines for NTDs. However, target-based programs require the availability of purified recombinant protein, the establishment of biochemical assays, and the availability of structural information to help illuminate hit discovery and optimization campaigns. Additionally, there is a chronic shortage of well-validated targets for NTD-causing organisms that could feed and sustain a robust drug discovery pipeline that is ready to respond to the certain emergence of resistant strains [7–9].

Deoxyhypusine synthase (DHS) is a promising target for small molecule-based therapies against NTDs. DHS catalyzes the NAD^+^-dependent transfer of the 4-aminobutyl moiety from the polyamine spermidine to a specific lysine residue in eukaryotic translation factor 5A (eIF5A) [10]. This reaction is the first step in the biosynthesis of hypusine in eIF5A. In a second reaction, production of mature, active eIF5A is carried out by deoxyhypusine hydroxylase (DOOH). In eukaryotic cells, mature eIF5A is required for translation elongation and termination, and essential for cell viability [11]. Likewise, DHS is thought to be essential in all eukaryotes [12], and gene knock-out experiments have shown DHS function to be essential for *Plasmodium falciparum*, *Leishmania donovani* and *Trypanosoma brucei* [13–15].

Most eukaryotic DHS proteins are functional homotetrameric enzymes. Crystal structures of human (Hs)DHS revealed that homotetramers have two pairs of identical active sites, and each active site is formed by residues from two distinct DHS protomers. The N-terminal region of each protomer in a DHS dimer reaches out and blocks the entrance of one of the two active sites in the adjacent homodimer. The sequence for this N-terminal region is widely conserved amongst various eukaryotic DHS proteins and is known as the “ball and chain” motif [16,17]. Mutational studies have also identified a series of structurally-conserved residues that are important for DHS function, including a conserved lysine residue that is essential for catalysis and forms a butylimine covalent intermediate during the DHS reaction [18].

As opposed to most eukaryotes, the genomes of trypanosomatid and *Entamoeba* species encode two DHS paralogs, known as DHSp and DHSc [15,19]. DHSp and DHSc associate to form a functional heterotretrameric enzyme. Both paralogs have the DHS fold, but DHSp lacks catalytic-important residues, including the conserved catalytic lysine residue. Consequently, DHSc/DHSp heterotetramers have two catalytically-active and two catalytically-dead sites. Both *Trypanosoma* and *Leishmania* DHS paralogs also lack the N-terminal ball and chain motif seen in other eukaryotic DHS enzymes.

Development of DHS inhibitors for the human enzyme is an on-going activity towards new anti-cancer therapies [20]. Similar efforts for DHS counterparts from pathogenic organisms have been hampered by a lack of structural information and compound screening assays. Further, the cellular roles and regulatory mechanisms of DHS enzymes from NTD-causing organisms, such as *L*. *major* and *B*. *malayi*, are not completely understood.

We have chosen BmDHS and LmDHS as candidate drug targets because both are suggested by the Tropical Disease Research (TDR) targets database (http://tdrtargets.org/). This database is an online resource to facilitate the prioritization of targets for drug development focused on pathogens causing neglected human diseases [21,22]. Furthermore, BmDHS is expressed in the adult stages of the parasite [23], being this very important since the adult worms are the cause of much of the pathology associated with lymphatic filariasis [24,25]. Additionally, BmDHS is likely to be essential for the survival of *B*. *malayi* in the human host [24]. On the other hand, in the case of *Leishmania*, both DHS counterparts show a low degree of sequence homology compared to human DHS (P49366) [26] and regardless of conservation of some of the active site amino acid residues between the human and leishmanial DHS, a potent inhibitor of human DHS, N1-Guanyl-1,7-diaminoheptane (GC7), had little inhibitory effect on *L*. *donovani* proliferation [14], indicating there is a difference between those enzymes that could be exploited to design new inhibitors [27].

Here, we obtained the crystal structure of NAD^+^-bound *B*. *malayi* (Bm)DHS and adapted a high-throughput screen (HTS)-ready biochemical assay to identify new inhibitors for this enzyme. Although we could not produce recombinant *L*. *major* (Lm)DHS in bacteria, we developed a yeast-based functional complementation system that can be used to find inhibitors of the *Leishmania* enzyme. Further, our data show that both LmDHSp and LmDHSc are required to complement DHS function in yeast. We expect our results to serve as the basis for future compound development campaigns aimed at chemically-validating DHS as a target for therapeutic interventions for *L*. *major* and *B*. *malayi* infections.

## Methods

### Plasmids, strains and media

For general cloning, we used *Escherichia coli* Mach-1 cells (Invitrogen, Carlsbard, USA). For protein production, we used BL21(DE3)-R3, a phage-resistant derivative of BL21(DE3) cells carrying plasmid pRARE2 (chloramphenicol resistant) [28]. Plasmids and *S*. *cerevisiae* strains used in this study are listed in Tables 1 and 2, respectively. The maintenance and cultivation of *S*. *cerevisiae* strains, composition and preparation of culture media and solutions, as well as techniques used in this study were performed according to standard protocols [29–31].

**Table 1:** Plasmids used in this study.

Plasmid	Description	Source
pSP-GM1	*2μ*; *URA3*; *TEF1pr*; *PGK1pr*; ampR	[32]
pCM188	*CEN*; *URA3*; *CYC1pr-tetO*_*2*_^1^; ampR;	[33]
pNIC28-Bsa4	pET expression vector; His_6_ tag in N-terminal; TEV protease cleavage site; sites for LIC cloning; “stuffer” fragment that includes the *sacB* gene; kanR; *T7pr*	[34]
pET-DUET1	Designed for coexpression of two target genes; ampR; *T7pr*	Merck Millipore (cat n° US171146-3)

^**1**^ *CYC1pr-tetO*_*2*_: tetracycline-repressible promoter

**Table 2:** *Saccharomyces cerevisiae* strains used in this study.

Strain	Genotype	Source
VZL1444	*MATα / MAT****a*** *can1pr*::*TDH3pr-E2Crimson*::*Hph*::*can1Δ*::*STE2pr-LEU2 / CAN1 lyp1Δ / LYP1 HYP2*::*natMX4 / HYP2 pdr5Δ*::*kanMX / PDR5 dys1Δ*::*HIS3 / DYS1 leu2Δ / leu2Δ his3Δ / his3Δ ura3Δ / ura3Δ met15Δ / met15Δ*	This work
VZL1462	*MAT****a*** *can1pr*::*TDH3pr-E2Crimson*::*Hph*::*can1Δ*::*STE2pr-LEU2 lyp1Δ pdr5Δ*::*kanMX ura3Δ his3Δ met15Δ*	This work
VZL1463	*MAT****a*** *can1pr*::*TDH3pr-E2Crimson*::*Hph*::*can1Δ*::*STE2pr-LEU2 lyp1Δ pdr5Δ*::*kanMX dys1Δ*::*HIS3 ura3Δ his3Δ met15Δ [pSP-GM1-LmDHSp/DHSc]*	This work
VZL1464	*MAT****a*** *can1pr*::*TDH3pr-E2Crimson*::*Hph*::*can1Δ*::*STE2pr-LEU2 lyp1Δ pdr5Δ*::*kanMX dys1Δ*::*HIS3 ura3Δ his3Δ met15Δ [pCM188-HsDHS]*	This work
VZL1400	*MAT****a*** *pdr5*::*HISMX dys1*::*kanMX his3Δ leu2Δ met15Δ ura3Δ [pCM188-BmDHS]*	[24]

### Cloning of DHS from *B*. *malayi* and *L*. *major*

Multiple fragments of BmDHS (Gene ID: 6098167), LmDHSc (Gene ID: 5654787) and LmDHSp (Gene ID: 5651301) were amplified by PCR and cloned into *E*. *coli* expression vector pNIC28-Bsa4, using ligation-independent cloning (LIC) [34–36]. Following this cloning strategy, recombinant proteins expressed from pNIC-Bsa4 vectors are fused to a N-terminal 6xHis tag, cleavable by treatment with the TEV (tobacco etch virus) protease. Detailed information about all constructs generated here, such as primer sequences, PCR template DNA, amplicon lengths and protein molecular weight, is available on S1 and S2 Tables. The DNA constructs used in this work for biochemical assays, yeast-based functional complementation system and structural determination were verified by sequencing (S1 File).

Small scale test expressions for BmDHS and LmDHSp/DHSc were performed in 1 mL bacterial cultures as described before [36]. Recombinant proteins were enriched from the clarified cell lysate using Ni-Sepharose resin (GE Healthcare Life Sciences), and analyzed by SDS-PAGE.

### Purification and crystallization of *B*. *malayi* DHS

For protein production, BL21(DE3)-R3-pRARE2 cells harboring plasmid pNIC28-Bsa4-BmDHS were grown in TB medium (supplemented with 50 μg.ml^−1^ kanamycin, 35 μg.ml^−1^ chloramphenicol) under agitation (140 rpm) at 37 °C until OD_600_ reached approximately 1.5. The cell culture was then cooled to 18 °C for 30 minutes and, after addition of isopropyl 1-thio-D-galactopyranoside (IPTG) to the media (0.2 mM final concentration), growth resumed at 18 °C overnight. Cells were harvested by centrifugation and pellets suspended in 2x lysis buffer (1 mL per gram of cells) (1x lysis buffer is 50 mM HEPES pH 7.5, 0.5 M NaCl, 10.0% (v/v) glycerol, 10 mM imidazole and 1 mM TCEP) supplemented with protease inhibitor cocktail EDTA-free (Merck Millipore; 1:200). Cells in 2x lysis buffer were flash-frozen in liquid nitrogen and stored at −80°C until use. After thawing cells were lysed by sonication on ice for 4 min (5 sec on, 10 sec off—amplitude = 35%) using a Sonics Vibra Cell VCX750 ultrasonic cell disrupter. Polyethyleneimine 0.15% was added to the cell lysate prior to centrifugation (45 min, 40,000 x*g*, at 4°C). The clarified lysate was applied onto a 5 mL HisTrap FF Crude column (GE Healthcare) connected to an AKTA pure protein purification system (GE Healthcare). BmDHS was eluted stepwise in lysis buffer with 300 mM imidazole. Removal of the hexahistidine tag was performed at 4 °C overnight using recombinant TEV protease while dialyzing against excess gel filtration buffer (20 mM HEPES, 500 mM NaCl, 1 mM TCEP, 5.0% [v/v] glycerol). BmDHS was further purified by reverse affinity in Ni-Sepharose followed by gel filtration (HiLoad 16/600 Superdex 200, GE Healthcare). BmDHS in gel filtration buffer was concentrated to approximately 11 mg.ml^−1^ using 30 kDa MWCO centrifugal concentrators (Millipore) at 4 °C. Protein concentration for the purified polypeptides was estimated by near UV absorbance at 280 nm using a NanoDrop (ThermoFisher) and Beer-Lambert equation A = ε x c x l, where ε is the BmDHS extinction coefficient (41370), c is the protein concentration in mol/L and l is the optical path length in cm. The protein was flash-frozen in a liquid nitrogen bath and stored at -80°C until use.

For crystallization, NAD^+^ and GC7 (diluted in gel filtration buffer) were added to purified BmDHS at 3-fold molar excess and incubated on ice for 1 h. The concentration of BmDHS in this mixture was returned to approximately 11 mg.ml−1 using centrifugal concentrators (30 kDa MWCO, Millipore) at 4°C prior to setting up 150 nL volume sitting drops at three ratios of the mixture composed by protein-NAD^+^-GC7 in relation to the reservoir solution (2:1, 1:1, or 1:2). Crystallization experiments were performed at 20 °C. Crystals were cryoprotected in reservoir solution supplemented with 25–30% glycerol before flash-freezing in liquid nitrogen for data collection. The best-diffracting crystals grew in a solution containing 20% PEG 3350 and 0.2 M ammonium citrate dibasic pH 5.0. Diffraction data were collected at the Diamond Light Source (DLS, Didcot OX11 0DE, United Kingdom) beamline I24.

### Structure solution and refinement

Diffraction data from BmDHS crystals were integrated using XDS [37] and scaled using AIMLESS from the CCP4 software suite [38]. Molecular replacement (MR) for BmDHS was performed with Phaser [39] using the human enzyme as template (PDB ID 1DHS) [16]. Refinement was performed in REFMAC5 [40]. Coot [41] was used for manual model building and refinement. NAD^+^ atoms were refined with full (1.0) occupancy. Structure validation was performed using MolProbity [42]. Structure factors and coordinates have been deposited in the PDB with the PDB ID 6W3Z (see Table 3). The final BmDHS model consisted of amino acids 5–245,351–359 for chain A; 28–360 for chain B; residues 9–360 for chain C, and 14–359 for chain D. Protein interfaces were calculated using the "Protein interfaces, surfaces and assemblies" (PISA) service at the European Bioinformatics Institute [43].

**Table 3:** Crystallographic data and refinement statistics for BmDHS-NAD^+^ complex.

Data collection	
**Crystal**	Native
PDB ID	6W3Z
X-ray source	DLS I24
Wavelength (Å)	0.9686
Space group	P 1 2_1_ 1
Cell dimensions	
*a*, *b*, *c* (Å)	69.753, 136.320, 75.883
α, β, γ (^o^)	90.00, 92.92, 90.00
Resolution (Å)	29.41–2.30 (2.36–2.30)
No. of unique reflections*	62,547 (4,609)
R_merge_ (%)	11.2 (75.2)
Mean I/σI	5.4 (1.1)
Mean CC_1/2_	0.99 (0.57)
Completeness (%)	99.6 (99.7)
Redundancy	3.3 (3.4)
Refinement Statistics	
Resolution (Å)	29.41–2.30 (2.36–2.30)
R_work_ / R_free_ (%)	18.8 (29.7) / 21.6 (30.6)
No. of atoms / Mean B-factor (Å)	
Protein atoms	10,533 / 37.7
Solvent atoms	397 / 37.4
NAD atoms	132 / 85.3
RMSD bond lengths	0.0045 Å
RMSD bong angles	1.26°
Ramachandran plot (%)	
Favored	98.5
Allowed	1.5
Outliers	0

Data for the outmost shell are given in parentheses.

### Structural comparison between BmDHS and HsDHS active sites

To delineate the protein substrate-binding pocket, our crystal structure of BmDHS was superimposed onto the available coordinates of the crystal structure of spermidine-bound HsDHS (PDB ID 6XXK) [44] and onto the crystal structure of GC7-bound HsDHS (PDB ID 1RQD) [17] using Pymol (Schrödinger, Inc) [45]. The structural alignments were used for comparison between HsDHS and BmDHS active sites.

### Homology modeling of *L*. *major* DHS

A homology model of LmDHSc and LmDHSp was built based on the crystal structure of the ternary complex of the *T*. *brucei* proteins bound to NAD^+^ (PDB ID 6DFT) [19] using SWISS-MODEL “Hetero Target” mode [46]. In this homology model, the N-terminal extension unique to LmDHSp was omitted and the amino acid insertions unique to LmDHSc were modelled as coils. SWISS-MODEL reported quality scores for the final homology model were: GMQE = 0.61, and QMEAN = -4.41.

### Determination of oligomeric state of BmDHS

The oligomeric state of BmDHS in solution was determined through the application of the purified protein onto the Superdex 200 Increase 10/300 (GE Life Science). Three different runs were used to obtain the standard curve, in which two of them contained a standard mix of proteins and the other one contained blue dextran 2000 to obtain the void volume. Firstly, blue dextran was applied, followed by the first mix with catalase (232 kDa), conoalbumin (75 kDa), and carbonic anhydrase (29 kDa). The second mix was composed by aldolase (158 kDa), ovoalbumin (44 kDa), and Ribonuclease A (13.7 kDa), then, 900 μg of purified BmDHS was applied onto the column. The standard proteins and the blue dextran 2000 belong to the low and high molecular weight gel filtration calibration kits (GE Life Sciences).

The proteins were solubilized in gel filtration buffer (20 mM HEPES pH 7.5, 300 mM NaCl, 5% Glycerol, 0.5 mM TCEP). The AKTA 25 L system flow was set up to 0.4 mL.min^-1^ and one milligram of each standard protein were loaded in a 500 μL injection. The elution volumes were used to calculate Kav values of each protein according to the following equation: Kav = (VE-V0) / (VC-V0), where VE, V0, and VC are the elution, void, and column volumes respectively. Kav values were plotted against the log of the molecular mass of the proteins to calculate the curve equation and discover the BmDHS mass in solution.

### Enzymatic assay for BmDHS

DHS activity was measured by following the increase in NADH fluorescence generated by the DHS-dependent reduction of cofactor NAD^+^ [47,48] using a CLARIOstar plate reader (BMG Labtech) set at the following wavelengths: excitation—355 ± 15 nm; emission—445 ± 20 nm. Measurements were recorded every 50 s following the start of the reaction for 50 min in total. Reactions were set at room temperature in Corning 384-well plates (Corning) with a final volume of 50 μL. All reactions were performed in buffer containing 0.2 M glycine / NaOH, pH 9.2; 5% glycerol; and 1 mM dithiothreitol (DTT, prepared fresh). We used this pH because it was the one that provided the highest activity for BmDHS (S1 Fig). In addition, this pH was also used in enzymatic assays with DHS from other species [14,47,49,50]. For the NAD^+^ titration experiment, final concentrations for reaction components were: spermidine—0.56 μM; BmDHS—100 nM (monomer); and NAD^+^—ranging from 0.195 to 400 μM. For the spermidine titration experiment, final concentrations for reaction components were: NAD^+^ - 132 μM; BmDHS—100 nM (monomer); and spermidine—ranging from 0.0005 to 1 μM. For the GC7 inhibition experiment test, final concentrations for reaction components were: spermidine—0.56 μM; NAD^+^ - 132 μM; BmDHS—75 nM (monomer); and GC7—ranging from 0.00017 to 10 μM. All reactions components were incubated for 1 h at room temperature prior to the addition of spermidine or NAD^+^ to start the reaction. Initial velocities at varying NAD^+^ or spermidine concentrations were estimated from reaction progress curves by least square fitting of the linear phase of the reaction. The dependence of the initial velocities on substrate or cofactor concentration was fitted to a hyperbola, Y = Vmax*X/(Kobs + X), where Vmax is the maximum enzyme velocity, X is the substrate concentration and Kobs is the substrate concentration needed to achieve a half-maximum enzyme velocity. Half-maximal inhibitory concentrations (IC_50_) were calculated by fitting the data to the equation: Y = Bottom + (Top-Bottom)/(1+10^((LogIC50-X)*HillSlope)), where Bottom and Top are plateaus in the units of the reaction rate, IC50 is the concentration of agonist that gives a response half away between Bottom and Top, X is the log of GC7 concentration and HillSlope describes the steepness of curves and it was used in this case a standard value of -1. Data points for individual experiments were collected in duplicates. All experiments were repeated twice. Data analysis employed GraphPad PRISM (version 6.00 for Windows).

### Functional complementation assays

#### Cloning in yeast expression vector

Synthetic genes encoding human (Hs)DHS (Gene ID: 1725) and BmDHS (Gene ID: 6098167) were codon-optimized for expression in *S*. *cerevisiae* (Geneart) and sub-cloned into the *BamH*I-*Pst*I sites of pCM188 [33] to produce pCM188-HsDHS and pCM188-BmDHS, respectively. Following this cloning strategy, the expression of heterologous genes is under control of a strong constitutive promoter *CYC1pr* [51]. Genes for both *L*. *major* paralogs (LmDHSc and LmDHSp) were PCR-amplified from *L*. *major* genomic DNA. LmDHSc was cloned into the *BamH*I-*Hind*III sites, and LmDHSp was cloned into the *Not*I-*Sac*I sites of pSP-GM1 [32], generating pSP-GM1-LmDHSc and pSP-GM1-LmDHSp, respectively. Alternatively, both LmDHS paralogs were cloned into a single pSP-GM1 plasmid, using the restriction sites cited above, and generating construct pSP-GM1-LmDHSp/DHSc. Using this cloning strategy, expression of LmDHSc and LmDHSp is under control of constitutive promoters *PGK1pr* and *TEF1pr*, respectively.

#### Yeast transformation and haploid selection

To generate DHS-complemented strains, the transformation of the heterologous constructs (pCM188-HsDHS, pCM188-BmDHS, pSP-GM1-LmDHSc, pSP-GM1-LmDHSp or pSP-GM1-LmDHSp/DHSc) was performed into a yeast strain heterozygous for a deletion of the *DYS1* yeast gene (encodes the DHS from *S*. *cerevisiae*, therefore herein called ScDHS) and heterozygous for *pdr5* (*pdr5Δ*::*kanMX*). *PDR5* encodes for *S*. *cerevisiae* most abundant drug export pump, hence its deletion generally results in an intracellular accumulation of test compounds [24]. After the transformation, these heterozygous strains harboring the heterologous constructs were sporulated and derived haploids were selected in the appropriated medium [31] for further assays.

Briefly, the following heterologous constructs: pCM188-HsDHS, pSP-GM1-LmDHSc, pSP-GM1-LmDHSp or pSP-GM1-LmDHSp/DHSc were transformed into diploid strain VZL1444 (Table 2). This diploid strain harbors a knockout of ScDHS, deleted by the insertion of the genetic marker *HIS3* (*dys1Δ*::*HIS3*), serving as a positive selection marker, since the strain will grow in media lacking histidine. The strain should be diploid considering the *DYS1* gene is essential in *S*. *cerevisiae*, requiring a wild-type allele. This strain also has other genes that can be used for selection of haploids (recessive markers) after mating and sporulation: *CAN1* and *LYP1* genes. *CAN1* and *LYP1* encode, respectively, arginine and lysine plasma membrane permeases and their deletions confer canavanine and thialysine resistance, respectively. The query strain also has the *STE2pr*-*LEU2* reporter, which promotes the expression of the *LEU2* gene only in haploid strains of mating type **a** (*MAT****a***). Therefore, the plating in synthetic medium lacking leucine, lysine and arginine, and complemented with canavanine and thialysine ensures the selection of haploids [52].

#### Yeast spot assay

The strains were grown in 5 mL of appropriate selective medium at 30°C until reaching approximately 1-2x10^7^ cells.mL^-1^. Then the cells were collected by centrifugation at 3,000 x*g* for 7 min and resuspended to the final concentration of 2.5x10^8^ cells.mL^-1^. After this procedure, 100 μL of this suspension were transferred into a 96 well microplate and 5 ten-fold serial dilutions were carried out. A volume of 4 μL of the original suspension and the dilutions were plated on the media of interest. The plates were then incubated at 30°C for 3 days. This experiment was conducted in triplicate comprising three independent experiments.

#### Western blot analysis

To assay the levels of hypusination, the yeast strains were grown overnight and then diluted to OD_600nm_ of 0.1 and grown to log phase at 30°C in 5 mL of culture. Subsequently, the cells were harvested by centrifugation at 4°C and stored at -80°C. Pellets were resuspended in 100 μL of lysis buffer (200 mM Tris/HCl, pH 7.0; 2 mM DTT; 2 mM EDTA; 2 mM PMSF and 5 μg.mL^-1^ of pepstatin, leupeptin, aprotinin and chymostatin) and lysed by agitation in Fast Prep equipment (three agitation cycles of 20 s) with glass beads. The cells were clarified by centrifugation at 20,000 *×g* for 15 min at 4°C and the supernatant was collected. Protein quantification was performed using DC Protein Assay (Bio Rad) against a bovine serum albumin (BSA; Sigma Aldrich) calibration curve. An amount of 7.5 μg of total protein were resolved on a 12% SDS-PAGE gel and transferred to nitrocellulose by a semi-dry transfer system (Bio Rad).

The immunoblot analysis was performed using a rabbit polyclonal anti-eIF5A (yeast) antibody [53] at a 1:10,000 dilution, a rabbit polyclonal anti-hypusine antibody (Millipore) [54] at a 1:2,000 dilution and a rabbit polyclonal anti-Rpl5 (yeast) antibody at a 1:25,000 dilution (loading control) [55], followed by anti-rabbit secondary antibody at a dilution of 1:20,000 (Sigma Aldrich). The proteins of interest were detected using a chemiluminescence detection system (ECL, GE Healthcare) on a Li-Cor C-Digit Blot Scanner.

### Growth analysis of DHS-complemented yeast strains in the presence of the drug GC7

The growth of DHS-complemented *S*. *cerevisiae dys1Δ* strains in the presence or absence of DHS inhibitor GC7 was monitored by optical density. Cells were grown at 30°C in 5 mL of culture media until stationary phase. Cells were then diluted in selective medium to an initial OD_600nm_ of 0.2 (approximately 1x10^6^ cells.mL^-1^) and a final volume of 150 μL in 96-well flat-bottom microplates (SPL—code 004393). The media used to dilute the cells contained either GC7 (at 1 mM final concentration diluted in acetic acid) or the acetic acid vehicle only (at 0.1 mM final concentration). Media for both groups contained aminoguanidine (1 mM final concentration) to prevent degradation of GC7 by monoamine oxidases [56]. Absorbance values (OD_600nm_) were acquired every 15 minutes for 15 h using a plate reader (Infinite 200 Pro, TECAN). The data were normalized by subtracting the OD_600nm_ value obtained for the GC7-treated or untreated condition by its respective background absorbance (medium plus compounds) values. These normalized data were converted to simple moving average, using four points, and these data was analyzed using Grofit package written in R, based on the model-free spline method [57]. A smoothed spline was fitted and growth scores were generated by calculating the maximum exponential growth rate for each replicate multiplied by the yield of the replicate (maximum OD600nm—minimum OD_600nm_) and then dividing this result by the lag phase duration [24,57]. Finally, a relative score for each strain was determined by the quotient of the score obtained for the treated condition and the score obtained for the untreated condition. A Student’s t-test (p < 0.05) was used to evaluate the differences in growth score for individual strains in the presence or absence of GC7. Differences in growth scores between strains were evaluated using a Tukey test (p < 0.05).

### Cell viability assay of DHS-complemented yeast strains after GC7 treatment

The DHS-complemented yeast strains were grown as described in the section “Growth analysis of DHS-complemented yeast strains in the presence of the drug GC7”. Also, the wild type strain (ScDHS) was treated with 10 mM H_2_O_2_ as a positive control of decrease of yeast cell viability [58]. The cell viability was determined by staining with methylene blue, as reported previously [58]. After growth of cell cultures, 140 μL of cultures were washed and resuspended in phosphate-buffered saline (PBS; 0.01 M pH 7.0) to approximately 10^8^ cells.mL^-1^, then 20 μL of the cell suspensions were mixed with 20 μL of methylene blue (0.1 mg. mL^−1^ stock solution, dissolved in a 2% dihydrate sodium citrate solution) and incubated for 5 min at room temperature. Subsequently, cells were examined under a light microscope in a Neubauer chamber. The number of stained (non-viable cells) or unstained (viable cells) were counted to a total of at least 320 cells per condition from two biological replicates [58,59]. The percentage of viable cells was calculated dividing the number of live cells by the total number of cells, multiplied by 100 [59]. A Student’s t-test was used to evaluate the differences in cell viability for each strain in the absence or presence of GC7.

## Results

### Cloning, expression and purification of *Brugia malayi* DHS

Currently, there is a lack of information on the biochemistry and structure of DHS proteins from nematode worms. To enable DHS from the filarial nematode worm, *B*. *malayi*, as a potential target for structure-based drug development programs, we sought to clone the gene encoding this enzyme and produce it recombinantly in *E*. *coli* cells. Previous genetic analysis of the gene encoding the DHS enzyme from the domestic silk moth (*Bombyx mori*) identified *bma-DHPS-1* (Gene ID: 6098167) as its counterpart in the genome of *B*. *malayi* [60].

**Fig 1:**
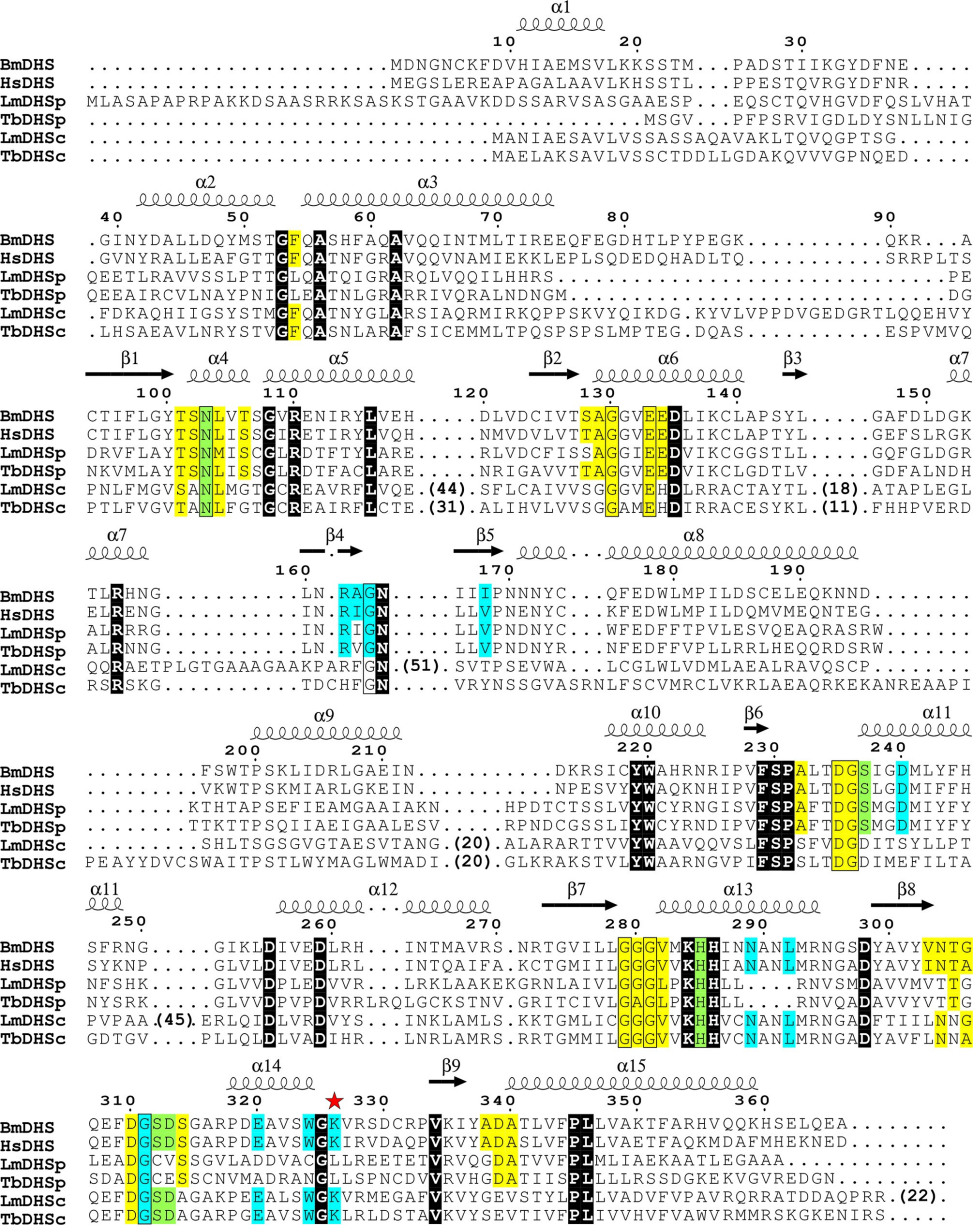
Structure-based sequence alignment of BmDHS, HsDHS, LmDHSp, LmDHSc, TbDHSp, and TbDHSc. Residues indicated by a light-colored background participate in binding to NAD^+^ (yellow) spermidine/GC7 (blue), or both ligands (green). Background coloring for LmDHSp/DHSc follow that of the *T*. *brucei* proteins. Residues indicated by a black background or framed in a box are conserved in all DHS proteins analyzed here. The catalytic lysine is marked with a red star. Gaps are indicated by black dots. For clarity, large gaps in LmDHSc were omitted and their lengths are indicated in parenthesis. The secondary structure (α-helices and β-sheets), and the numbering shown in the top line are for BmDHS. Protein sequences and structures used in the alignment were: BmDHS (UniProt ID A0A0J9XTC4; PDB ID 6W3Z), HsDHS (UniProt ID P49366, PDB ID 1DHS), LmDHSp/DHSc (UniProt IDs Q4QD19 / Q4Q3H5), and TbDHSp/DHSc (UniProt IDs Q4GZD1 / Q38BX0, PDB ID 6DFT). Structural alignment by PROMALS3D [61].

To increase the chances of producing the heterologous recombinant protein in a soluble form, we adopted a similar strategy to that of The Structural Genomics Consortium [35]. We designed primers to generate four different PCR amplicons from the *bma-DHPS-1* gene template, each one encoding different *B*. *malayi* (Bm)DHS proteins having disparate N- and C-terminal boundaries. These boundaries were selected based on a structure-based sequence alignment between the filarial worm enzyme and its closest counterpart in the Protein DataBank (PDB), the human (Hs)DHS enzyme (PDB entry ID 1DHS) [16] (Fig 1 and S1 and S2 Tables). Small scale test-expression of the four different BmDHS constructs, revealed that three of them (BmDHS-cb001, BmDHS-cb002 and BmDHS-cb003) could be produced in a soluble form in the bacterial strain used here (BL21(DE3)-R3-pRARE2) [35], including the one construct encoding the full-length enzyme (S2 Table and S2 Fig).

Next, we scaled-up production of the full-length recombinant BmDHS. The protein was purified to homogeneity using a combination of immobilized metal ion affinity (IMAC) and gel filtration chromatography (S2 Fig). The recombinant BmDHS produced here had an additional N-terminal serine residue compared to the wild-type protein encoded by the *bma-DHPS-1* gene. This additional residue was introduced by our cloning strategy and left over after cleavage of the N-terminal poly-His tag with TEV protease (see Methods for further details). The purified recombinant BmDHS had the expected molecular weight (41,083.5 Da), as determined by LC-MS (S3 Fig). We estimated a final yield of approximately 2.8 mg of purified BmDHS per liter of bacterial culture used.

Mammalian DHS is a tetramer in solution. We used analytical gel filtration chromatography to investigate if the recombinant BmDHS protein produced here also had a similar quaternary structure. Purified recombinant protein had an apparent partition coefficient equivalent to that of a globular protein of approximately 165 kDa. This value is in excellent agreement with the expected size of a BmDHS tetramer (approximately 164 kDa) (S3 Fig).

### Cloning and expression of *Leishmania major* DHSp and DHSc proteins

Given our success in producing recombinant BmDHS in bacterial cells, we sought to expand our efforts to the counterpart proteins from *Leishmania major*. The genome of *L*. *major* encodes two DHS paralogs—LmDHSp is encoded by gene LmjF.20.0250 (GeneID 5654787), and LmDHSc by gene LmjF.34.0330 (GeneID 5651301).

As for BmDHS above, we attempted to produce both LmDHS paralogs recombinantly using *E*. *coli* cells. For each LmDHS, we successfully cloned four different constructs into our modified pET-based expression vector. The boundaries for these constructs were based on the crystal structure of the NAD^+^-DHSp-DHSc ternary complex from *T*. *brucei* (PDB ID 6DFT) [19] (Fig 1, and S1 and S2 Tables). Nevertheless, none of these constructs resulted in the production of soluble protein from bacterial cells. Concomitant expression of both LmDHS paralogs also failed to produce soluble protein in bacteria (S4 Fig).

### Structure of BmDHS bound to NAD^+^

Structural information can accelerate chemistry efforts in target-based drug discovery campaigns. Currently, there is no structural information available for nematode DHS proteins. We thus determined the crystal structure of full-length, recombinant BmDHS (residues 1–366) bound to cofactor NAD^+^ to a resolution of 2.3 Å. We used the human enzyme (PDB ID 1DHS) [16] as a search model in molecular replacement to solve the crystallographic phase problem (Table 3). The crystal structure of BmDHS revealed a similar structure and quaternary organization to its human counterpart, as expected from the high identity levels between these two proteins (~60%) and our results from analytical gel filtration above.

**Fig 2:**
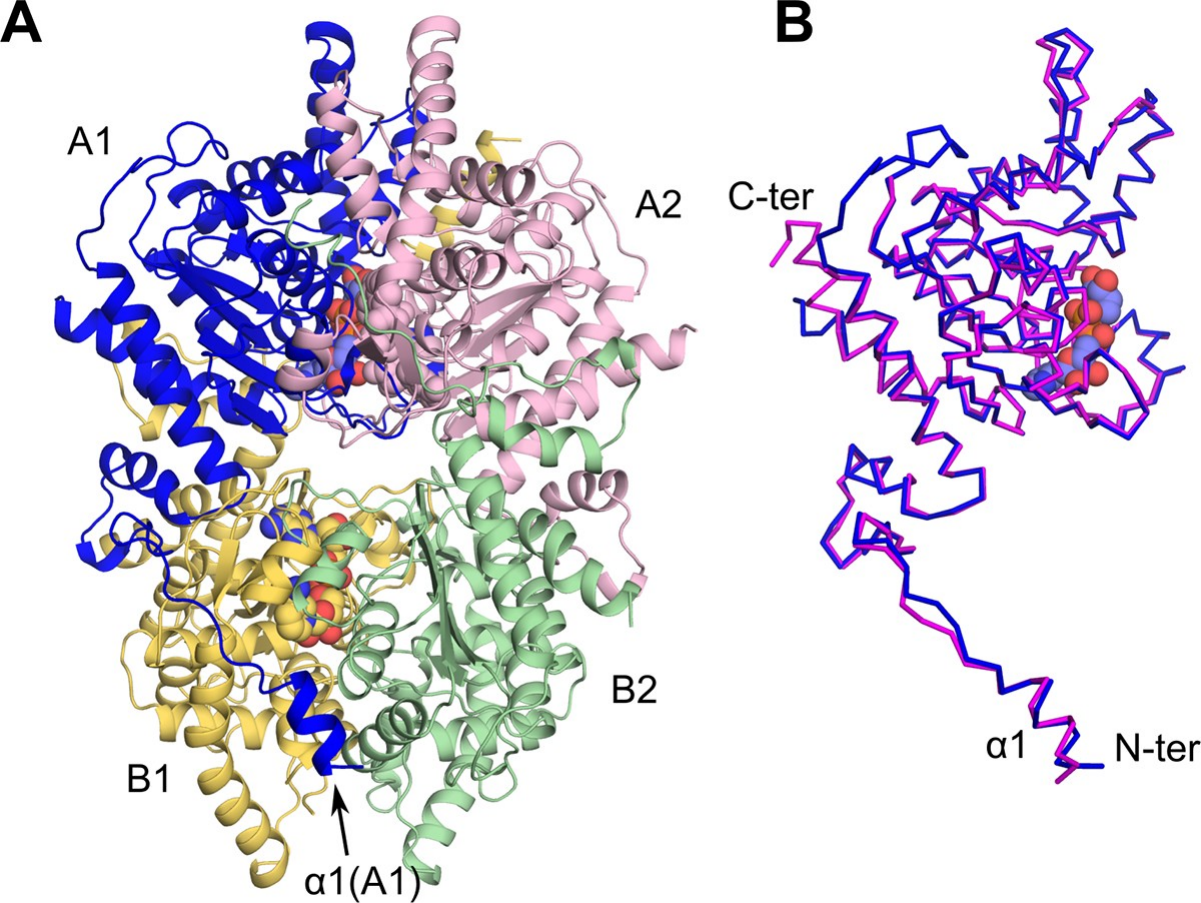
Overall structure of NAD^+^-bound BmDHS. (A) Cartoon representation of the BmDHS tetramer. Individual protomers within the tetramer are depicted in different colors (A1—blue, A2—pink, B1—yellow, and B2—green). NAD^+^ molecules are shown in a sphere representation. (B) Superposition of a BmDHS protomer (A1 in blue; same orientation as in panel A) onto its human counterpart (magenta).

Within the crystal asymmetric unit, four BmDHS protein chains (hereafter referred as A1, A2, B1, and B2—see Fig 2A) organized themselves into a tetramer. Individual protomers had very similar structures (root mean square deviation, r.m.s.d. ≤ 0.30 Å). The most notable structural differences amongst individual BmDHS protomers located to the protein N-terminal region, which is known to be highly flexible [16,17]. For example, no electron density was observed for the first 28 residues of protomer A2. Likewise, protomers of human *and B*. *malayi* DHS shared a high degree of structural similarity (r.m.s.d, 0.50 Å), and the most notable structural differences between the two proteins located to the N-terminal region (Fig 2B).

**Fig 3:**
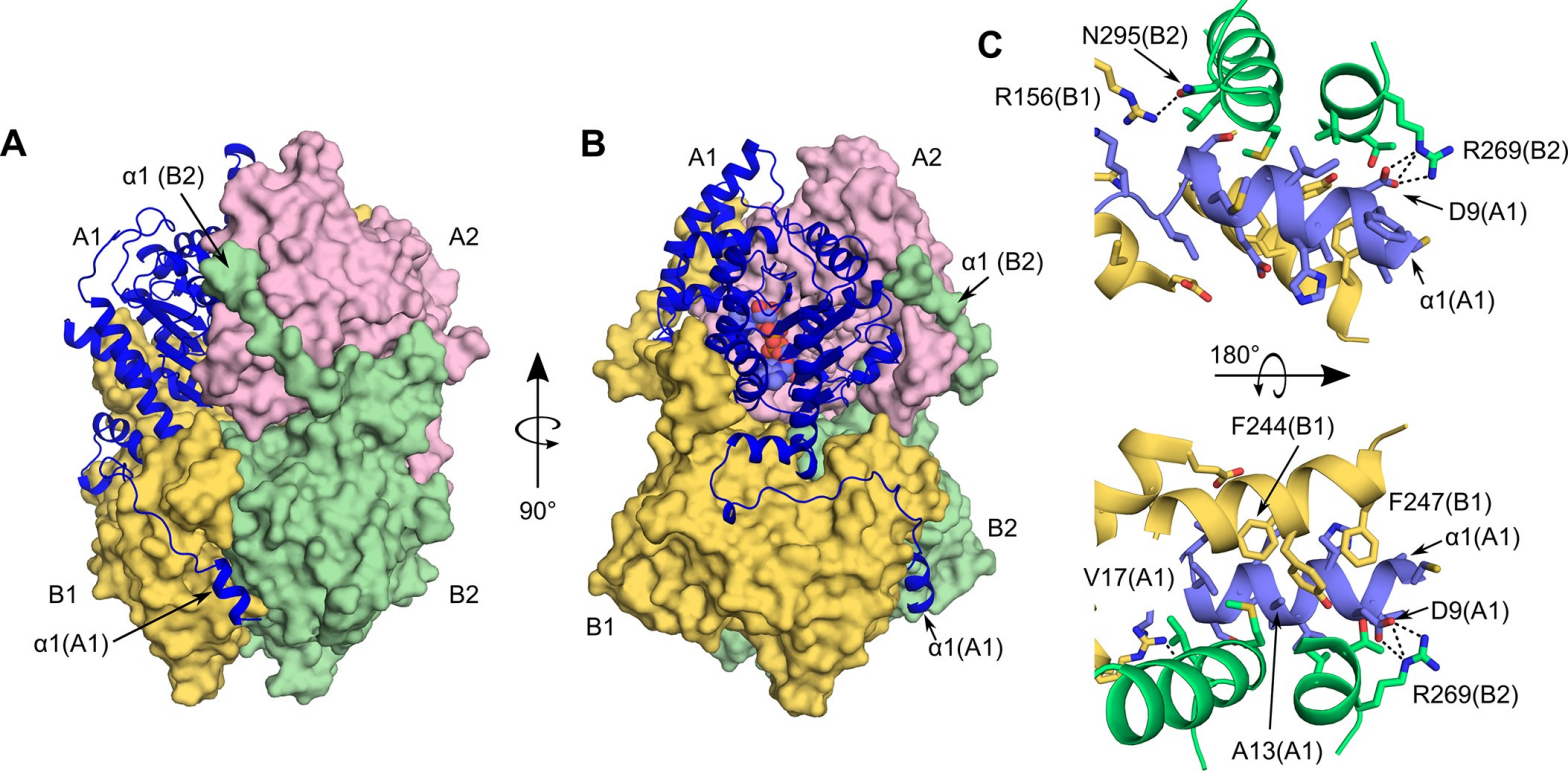
Interprotomer contacts within a BmDHS homotetramer. (A, B) Molecular surfaces for protomers A2, B1, and B2 are shown in the same colors as in Fig 2A. Protomer A1 is depicted as a blue cartoon. The position of the N-terminal α1 is indicated. (C) Interactions between the N-terminal α1 from protomer A1 and nearby (4 Å cut-off) residues from protomers B1 and B2. Hydrogen bonds are depicted as dashed black lines.

As seen for the human enzyme, the BmDHS tetramer had four active sites. Within a DHS tetramer, two active sites were formed by residues from protomers A1 and A2, and two by residues in protomers B1 and B2. The interface area for protomers contributing residues to the same active site was quite extensive and totaled ~2,500 Å^2^ (Fig 3A and 3B). NAD^+^ molecules occupied three out of the four active sites of the tetramer (see below).

The structure of the human DHS revealed that access to the enzyme active site is controlled by a “ball-and-chain” mechanism [16,17]. Likewise, in the BmDHS crystal structure, the entrance to the two active sites formed by protomers A1 and A2 were blocked by the N-terminal helices (α1, the “ball”) from protomers B1 and B2. Due to poor electron density, the equivalent N-terminal region from protomer A2 was omitted from the final protein model. Thus, one of the active sites formed by protomers B1 and B2 was open to the solvent. The other active site formed by protomers B1 and B2 was blocked by α1 from protomer A1. Interprotomer contacts facilitated by α1 were mostly of a hydrophobic nature (Fig 3C). The only polar contacts observed were those between side chain atoms of Asp9 in α1 and Arg269 from a different protomer.

In addition to the contacts described above for α1, the observed interface area between protomers that contributed residues to different active site was quite extensive. For example, the interface area between protomers A1 and B1 totaled ~3,500 Å^2^, and that between A1 and B2 ~650 Å^2^. Altogether, interprotomer contacts buried ~12,600 Å^2^ of surface area (Fig 3A and 3B). Such an extensive interface area, together with our analytical gel filtration analysis (S3 Fig), strengthen the idea that, as shown previously for DHS from other organisms [62–64], the biologically relevant assembly for the filarial worm DHS is a homotetramer.

### BmDHS active site

The structure of BmDHS obtained here allowed us to define the enzyme active site. Within the BmDHS tetramer, all four active sites had similar architectures. Below we describe one of them in detail—the active site formed by protomers A1 and A2 and whose entrance was blocked by the α1 from protomer B1. Given the structural conservation amongst individual protomers, similar conclusions can be drawn for all four active sites within the BmDHS tetramer.

**Fig 4:**
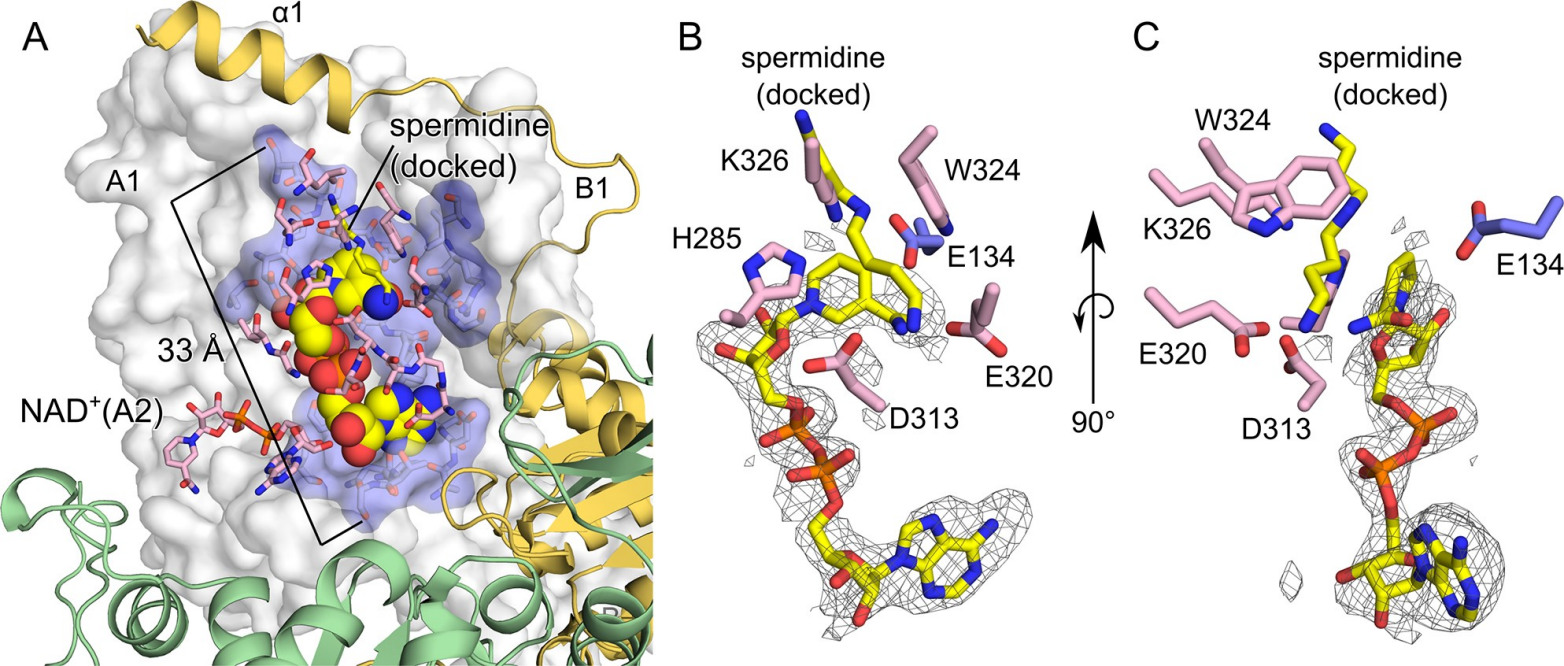
Details of the BmDHS active site. (A) Protomer A1 is shown as a white surface, with residues within a 4 Å radius of the NAD^+^ cofactor (spheres) shown as blue sticks and highlighted by pale blue surface. Residues in protomer A2 within a 4 Å radius of the NAD^+^ cofactor bound to protomer A1 are shown as pink sticks. The NAD^+^ cofactor bound to protomer A2 is also shown as pink sticks. Protomers B1 (yellow) and B2 (green) are shown as cartoon. (B, C) Close view showing catalytically-important residues within BmDHS active site. Polder OMIT map [65] for NAD^+^. The grey mesh represents the (*mF*_obs_-*DF*_model_) OMIT difference density contoured at 3.0 σ. Spermidine (yellow stick) was docked following the superposition of the crystal structure of BmDHS onto the crystal structure of spermidine-bound HsDHS (PDB ID 6XXK) [44] using Pymol (Schrödinger, Inc).

As seen for the human DHS, the *B*. *malayi* enzyme active site had a funnel-like shape. The wider end of the active site was closer to the protein surface and was blocked by α1 from protomer B1. The narrower end of the active site was deep under the protein surface (approximately 30 Å, from the side chain phenyl ring of Phe244 at the active site entrance to Cα atom of Asn304 at its bottom). Within BmDHS active site, the NAD^+^ molecule was oriented in such a manner that its adenine ring was accommodated by protein residues at the bottom of the funnel, whereas the cofactor nicotinamide ring pointed towards the active site entrance (approximately 15 Å to Phe244 phenyl ring). S3 Table lists residues in BmDHS with atoms within a 4.0 Å radius of atoms from the NAD^+^ cofactor. The diphosphate group and the two ribose moieties from the NAD^+^ molecule found in the second active site formed by protomers A1 and A2 defined one of the walls of the active site under analysis here (Fig 4 and S3 Table).

Although present in the crystallization cocktail, no electron density was observed for the DHS inhibitor GC7 in our BmDHS crystals. In our BmDHS crystal structure, the entrance to the active site was blocked by the N-terminal helix (α1, the “ball”), and that might have prevented the binding of GC7 to BmDHS in the crystallization cocktail. Similarly, GC7 could not be located in electron density maps of crystals of the human enzyme that had blocked enzyme active sites [16].

To confirm the reaction mechanism of BmDHS, and to better delineate the protein substrate-binding site, we performed a simple, rigid body docking of spermidine, as seen in the HsDHS cocrystal structure (PDB ID 6XXK) [44], onto the active site of the *B*. *malayi* enzyme. These analyses suggested that the substrate can be accommodated in the active site of BmDHS without the need of major structural rearrangements (Fig 4). BmDHS residues within 4.0 Å of the docked spermidine molecule are listed in S3 Table. Together, residues in close proximity to NAD^+^ and spermidine defined the active site of BmDHS (S3 Table). The same results were obtained by docking GC7 instead of spermidine, as both GC7 and spermidine occupy the same pocket within the BmDHS structure (S5 Fig and S3 Table).

### BmDHS proposed reaction mechanism and interaction with eIF5A

The BmDHS crystal structure and the docking of spermidine onto this structure (Fig 4) suggested that the reaction mechanism for the *B*. *malayi* enzyme is similar to that suggested for the mammalian enzyme [66,67]. In the first step of the reaction catalyzed by DHS, the substrate spermidine is oxidized to dehydrospermidine, in an NAD^+^-dependent way [47,66]. In the crystal structure of BmDHS, the nicotinamide ring from the NAD^+^ cofactor was in close proximity to the docked spermidine molecule (Fig 4). This finding suggested that the NAD^+^ cofactor would be capable of abstracting a proton from the central secondary amine of the spermidine substrate. In the second step of the reaction, a conserved lysine residue attacks the oxidized spermidine forming an enzyme-butylimine covalent adduct and releasing propane-1,3-diamine (DAP) [47]. In BmDHS, this conserved lysine residue is Lys326. In the worm DHS structure, Lys326 is in close proximity to the docked spermidine molecule and in a good position to attack the central amino group of dehydrospermidine. Other residues found important for catalysis in mammalian DHS, such as Glu137, His288, Asp316, Glu323 and Trp327 (Glu134, His285, Asp313, Glu320 and Trp324 in BmDHS, respectively) are conserved between the human and filarial worm proteins and occupy structurally-equivalent positions within the active site of these two enzymes [18] (Fig 4 and S3 Table).

In the next steps of the reaction, the butylimine group is transferred to a conserved lysine residue within the eukaryotic translation initiation factor 5A (eIF5A) and NAD^+^ is regenerated within the DHS active site. In human (Hs) eIF5A, the target of hypusine modification is residue Lys50, which is located at the tip of a long, finger-like, loop [68]. The proper anchoring of eIF5A onto DHS is likely to require extensive contacts between the two proteins, as almost all of the N-terminal domain of HseIF5A (residues 20–80) must be intact for it to be modified by the human enzyme [69].

We used HseIF5A (UniProt ID P63241) in a BLAST search [70] to identify the worm counterpart of the human protein. *Brugia malayi* genome encodes a single protein (UniProt ID A0A0I9R327; GeneID 6106396) with high sequence identity to human (64%) and *S*. *cerevisiae* (58%) eIF5A proteins. Importantly, sequence identity levels between BmeIF5A and its counterparts in *S*. *cerevisiae* and humans were substantially higher at the protein N-terminal domain (~70 and 80%, respectively) (S6 Fig).

Taken together these analyses suggested that BmDHS has a similar structure and reaction mechanism to those established for the mammalian enzyme. Further, our results also indicated that the binding interface between eIF5A and DHS is likely to be similar for the worm, human and baker’s yeast proteins.

### Homology modelling of *Leishmania major* DHSp and DHSc proteins

As recombinant DHS paralogs from *L*. *major* were recalcitrant to recombinant production in bacteria, we turned to primary sequence analysis and homology modelling to provide a first glance on the structural features of LmDHSp and LmDHSc that might be important for their function. Currently, structural information is available for *T*. *brucei* (Tb)DHSp and (Tb)DHSc, which share between 40 to 55% sequence identity to the two *L*. *major* proteins, respectively. A primary sequence analysis revealed striking differences between DHS paralogs from *T*. *brucei* and *L*. *major*. Of note, LmDHSp has a single 44-amino acids insertion at its N-terminal that is not present in the *T*. *brucei* protein. This N-terminal extension is also not observed in the human and filarial worm homologs. By contrast, LmDHSc is 140-amino acids longer than TbDHSc, with large (≥15) amino acids insertions, and these are mostly located to the mid- and C-terminal regions of the protein. These amino acid insertions are also not observed in the human and worm enzymes (Fig 1).

On the other hand, DHSp and DHSc paralogs from *T*. *brucei* and *L*. *major* share important features with other DHS homologues. For example, DHSc homologues from both parasites share catalytically-important resides with DHS homologues, including the catalytic lysine residue (Lys329 in HsDHS, Lys326 in BmDHS, Lys418 in TbDHSc and Lys535 in LmDHSc). By contrast, in the DHSp proteins from *T*. *brucei* and *L*. *major* this catalytically-important lysine has been replaced by a leucine residue (Leu303 in TbDHSp and Leu344 in LmDHSp).

To further explore the unique features of the *L*. *major* proteins, we used SWISS-MODEL to build a homology model of both LmDHS paralogs based on the available structural information from *T*. *brucei* proteins—as seen in the crystal structure of the ternary complex of NAD^+^-TbDHSp/TbDHSc (PDB ID 6DFT) [19]. In this homology model, the N-terminal extension unique to LmDHSp was omitted and the amino acid insertions unique to LmDHSc were modelled as coils. SWISS-MODEL reported quality scores for the final homology model were: GMQE = 0.61, and QMEAN = -4.41. Homology models having QMEAN score ≤ 4.0 are considered to be of low quality. Nevertheless, in our LmDHS homology model, the conserved protein core and catalytic-important residues in LmDHSc, and the majority of the LmDHSp protomer displayed high local similarity with the target *T*. *brucei* DHS structure. On the other hand, the low-quality regions of the LmDHS homology model located to the large inserts present in LmDHSc are absent in the *T*. *brucei* protein (S7 Fig).

TbDHS paralogs have the same overall architecture of a human DHS monomer. However, in the TbDHS heterotetramer, DHSc and DHSp play distinct but complementary roles, whereas in the human and worm DHS homotetramers, all protomers are thought to perform identical, independent catalytic functions. We built our LmDHS heterotetramer to have the same architecture as the one observed for the *T*. *brucei* complex, in which DHSp and DHSc form a heterodimer and share one functional catalytic site and one catalytically dead site. LmDHSp and LmDHSc protomers are the structural equivalent of protomers A1 and B2, and A2 and B1, respectively, in the BmDHS structure reported here (S8 Fig).

Analysis of the resulting homology model suggested that both *T*. *brucei* and *L*. *major* DHS heterotetramers share conserved residues at structurally-equivalent positions within their catalytically active and dead sites (S3 Table and S9 Fig). It is thus likely that LmDHS heterotetramers, just like their counterparts from *T*. *brucei*, bind four NAD^+^ molecules, but have only two catalytically active sites. In the model, all four sites were open to the solvent. Both DHSc and DHSp seem to lack an equivalent structure to the N-terminal helix (the “ball”) seen in homotetrameric DHS enzymes. On the other hand, the unique amino acid inserts in LmDHSc do not have a structural counterpart in the *T*. *brucei* protein or in the human and worm DHS proteins. Our model suggested that two pairs of inserts in LmDHSc could potentially block the entrance of both catalytically competent (inserts α3-β1, and α5-β2) and catalytically dead (inserts β4-β5, and α11-α12) sites within a LmDHSp/DHSc heterodimer (Panel C in S8 Fig). In such case, these inserts would play an equivalent role to that of the N-terminal α-helix in homotetrameric DHS proteins. Nevertheless, as highlighted above, the LmDHS homology model obtained here is not accurate enough to infer the structure adopted by the amino acid inserts unique to the *Leishmania* protein. We expect the future structural characterization of LmDHSp/DHSc to illuminate the function of these unique regions.

### Establishment of a simple, HTS-ready biochemical assay for BmDHS

A robust and sensitive *in vitro* assay is a critical component of a target-based drug discovery platform. Ideally, biochemical assays for drug discovery campaigns should be low-cost and amenable to automation. These assays can be used to enable the screening of compound libraries and illuminate synthetic chemistry efforts. The conventional assay used to measure DHS activity relies on the incorporation of radioactivity from ^3^H-labelled spermidine into eIF5A. This assay has been shown robust and reliable, but cannot be readily adapted for the high throughput screening (HTS) of compound libraries. Recently, Park and colleagues described a non-radioactive assay that can measure the free NADH from the DHS partial reaction using an independent coupling reaction (NADH-Glo) [71]. This NADH-Glo coupled assay can be easily used for HTS campaigns but might be susceptible to artifacts that interfere with the luciferase-based coupled reaction [72,73]. We thus sought to establish an alternative enzymatic assay that could be used to complement the strengths of the two methods mentioned above.

**Fig 5:**
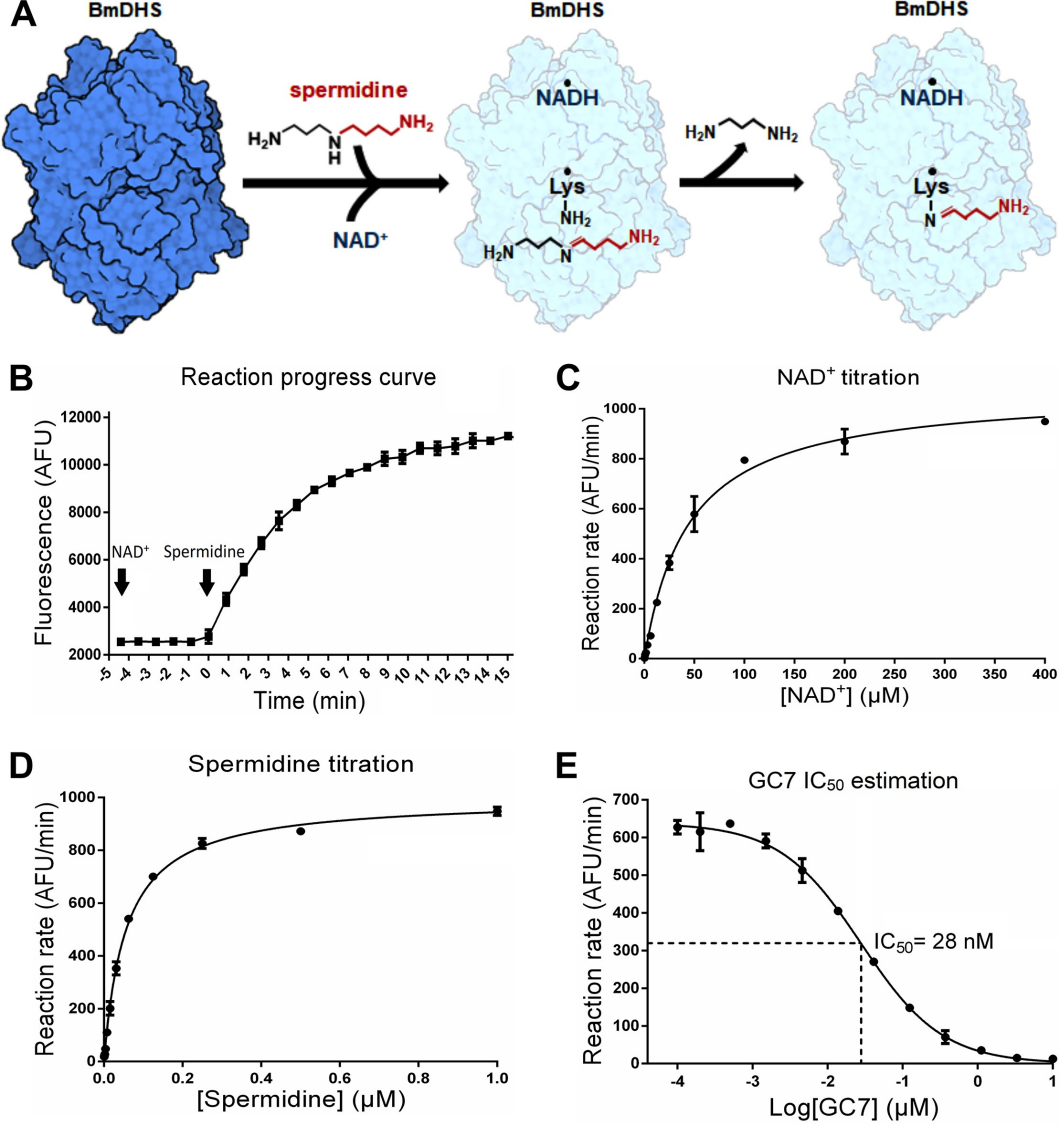
Partial reaction mechanism, establishment of a NAD^+^/NADH fluorescence enzymatic assay for the BmDHS partial reaction, and IC_50_ determination for GC7. (A) Proposed reaction mechanism for a single turnover catalyzed by BmDHS in the presence of NAD^+^ and spermidine. Maintenance of the covalent adduct between BmDHS Lys326 and the butylimine group from spermidine keeps the enzyme in an inactive form. In the absence of eIF5A, DHS is not regenerated. (B) Reaction progress curve demonstrating the generation of NADH by BmDHS. Fluorescence emission intensity after excitation at 355 ± 15 nm. The final component of the reaction was added to the plate at time 0 and the fluorescence intensity was measured during the indicated time. The reaction (50 μL) was composed of 100 nM BmDHS, 132 μM NAD^+^ and 0.5 μM spermidine. (C) Titration of NAD^+^. Variable concentrations of NAD^+^ (0.195 to 400 μM) and fixed concentrations of BmDHS (100 nM) and spermidine (0.56 μM) were used to carry out the experiment. (D) Titration of spermidine. Variable concentrations of spermidine (0.0005 to 1 μM) and fixed concentrations of BmDHS (100 nM) and NAD^+^ (132 μM) were used to carry out the experiment. (E) Determination of the IC_50_ for GC7. Variable concentrations of GC7 were used (0.00019 to 10 μM) and fixed concentrations of BmDHS (75 nM), spermidine (0.56 μM) and NAD^+^ (132 μM) were used to carry out the experiment. The estimated value for IC_50_ is shown in the figure. The individual points represent the mean ± standard error of experimental duplicates.

The biochemical assay reported here relies on the known ability of DHS to catalyze a partial reaction in the presence of NAD^+^ and spermidine resulting in the formation of the enzyme-butylimine intermediate and enzyme-bound NADH, even in the absence of the eIF5A precursor [67] (Fig 5A). Thus, in our assay, we follow the fluorescence from NADH (emission peak approximately 440 nm) generated in a single-turnover of the partial DHS reaction [47,67] (Fig 5A). Some of the advantages of this assay include the direct coupling of the fluorescent signal to the partial DHS reaction, the ease of miniaturization to fit an HTS format, and the reduced costs compared to those of the NADH-Glo alternative. On the other hand, the use of this assay in HTS campaigns is likely to require the use of follow-up assays, such as the NADH-Glo assay above, to rule out false-positive artifacts that may fluoresce at similar wavelengths [74].

To ensure this assay was indeed robust and sensitive, we followed NADH-produced fluorescence at increasing concentrations of purified recombinant BmDHS in the presence of excess cofactor NAD^+^ and substrate spermidine (132 and 0.5 μM, respectively). The resulting reaction progress curves allowed us to determine an enzyme concentration that produced a robust signal to noise ratio at the linear range of the reaction (Fig 5B). Based on these results, we chose to work with an enzyme (tetramer) concentration of approximately 25 nM.

To determine an appropriate, saturating concentration of NAD^+^ to use in the assay, we followed reaction progress curves in the presence of increasing concentrations of the cofactor (0.195 to 400 μM) and a fixed, excess concentration of the substrate spermidine (0.56 μM) (Panel A in S10 Fig). These curves were used to calculate the linear rate of NADH formation at various NAD^+^ concentrations (*v*_NAD+_). A plot of *v*_NAD+_ values versus NAD^+^ concentrations was used to establish the hyperbolic relationship of the single turn-over reaction with cofactor concentration (K_obs_,_NAD+_ 44 ± 3.2 μM) (Fig 5C). From these studies, we chose to use NAD^+^ at a fixed concentration of 132 μM.

We performed equivalent experiments to establish appropriate, saturating concentrations of BmDHS substrate spermidine (Panel B in S10 Fig). In these experiments, NAD^+^ was kept fixed at 132 μM and the linear rate of NADH formation was calculated for increasing spermidine concentrations (0.0005 to 1 μM) (*v*_spe_). A plot of *v*_spe_ values versus spermidine concentrations was used to establish the hyperbolic relationship of the single turn-over reaction with substrate concentration (K_obs_,_spe_ 0.056 ± 0.002 μM) (Fig 5D). From these studies, we chose to use spermidine at a fixed concentration of 0.56 μM.

Following up on these preliminary studies, we sought to show this assay can be used to estimate half-maximal inhibitory concentration (IC_50_) values for BmDHS inhibitors. In these assays, we employed GC7, a potent inhibitor of the mammalian DHS [56,75]. We used reaction progress curves to estimate the linear rate of NADH formation by BmDHS at increasing concentrations of GC7 (0.00019 to 10 μM), whilst maintaining fixed concentrations of NAD^+^ (132 μM), spermidine (0.56 μM), and enzyme (18.75 nM, tetramer). The resulting dose-response curve was used to estimate an IC_50_ value of 28 nM for GC7 (Fig 5E).

### Development of a *S*. *cerevisiae*-based assay for DHS activity

The yeast *S*. *cerevisiae* has been used as an important tool to understand the function of heterologous enzymes and for the search and study of small molecule inhibitors [76–81]. Functional replacement of essential genes in yeast by their counterparts from the target pathogenic organism has been successfully employed to support target-based drug discovery programs for proteins recalcitrant to recombinant production, such as LmDHSp/DHSc here. Moreover, replacement of the essential yeast gene with its human counterpart provides the means to identify small molecules that only affect the protein from the pathogenic organism, but not the host’s [24].

**Fig 6:**
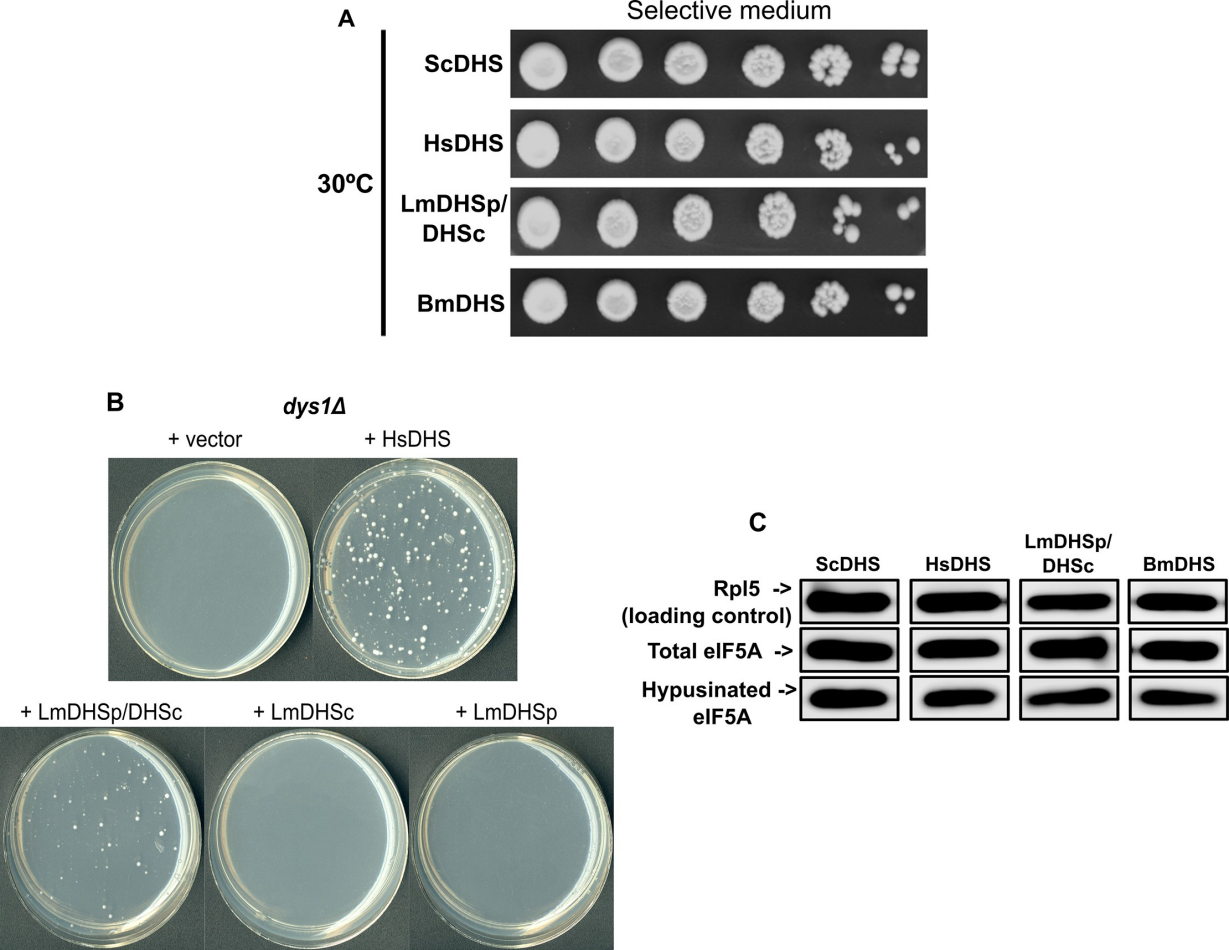
Functional replacement (complementation) of yeast deoxyhypusine synthase by orthologue enzymes from eukaryotic pathogens. (A) Ten-fold serial dilutions of the indicated strains (after haploid selection- see Material and Methods) were plated on selective media and grown at 30°C for 3 days. (B) Complementation of the yeast deoxyhypusine synthase deleted strain only by the two isoforms of LmDHS. The indicated strains were plated on selective media during haploid selection and grown at 30°C for 3 days. (C) Hypusination of eIF5A in the yeast strains complemented with the DHS from different organisms.

To generate such a system for DHS in *S*. *cerevisiae*, we produced mutant strains lacking the endogenous gene encoding ScDHS (*dys1*). To increase the sensitivity of our yeast DHS system to test compounds, we generated the *dys1Δ* mutant strain in a genetic background lacking Pdr5, the major drug export pump in *S*. *cerevisiae* [24]. In *S*. *cerevisiae*, hypusine modification of eIF5A by a functional DHS is required for growth [82,83]. As expected, following deletion of *dys1*, yeast cells could not grow, unless complemented by the ectopic expression of ScDHS (Fig 6A).

The eIF5A homologs from human, yeast, filarial worm, and *L*. *major* have high sequence identity levels (≥ 50%), especially at their N-terminal region (> 60%), which is required for recognition and modification by DHS [69] (S6 Fig). Thus, we reasoned that ectopic expression of a functional, heterologous DHS should rescue the lethal phenotype observed in the *dys1Δ* strain. In this context, separate epigenetic expression of LmDHSc or LmDHSp in *dys1Δ* mutants did not produce viable cells. On the other hand, concomitant expression of both LmDHSc and LmDHSp did rescue *dys1Δ* mutants, suggesting that both *L*. *major* DHS paralogs are required to complement the function of yeast deoxyhypusine synthase (Fig 6B). Similar experiments showed that ectopic expression of HsDHS and BmDHS also rescued *dys1Δ* mutants (Fig 6A). More importantly, ectopic expression of all three heterologous DHS resulted in hypusination of the endogenous yeast eIF5A, as shown by western blot analysis (Fig 6C).

**Fig 7:**
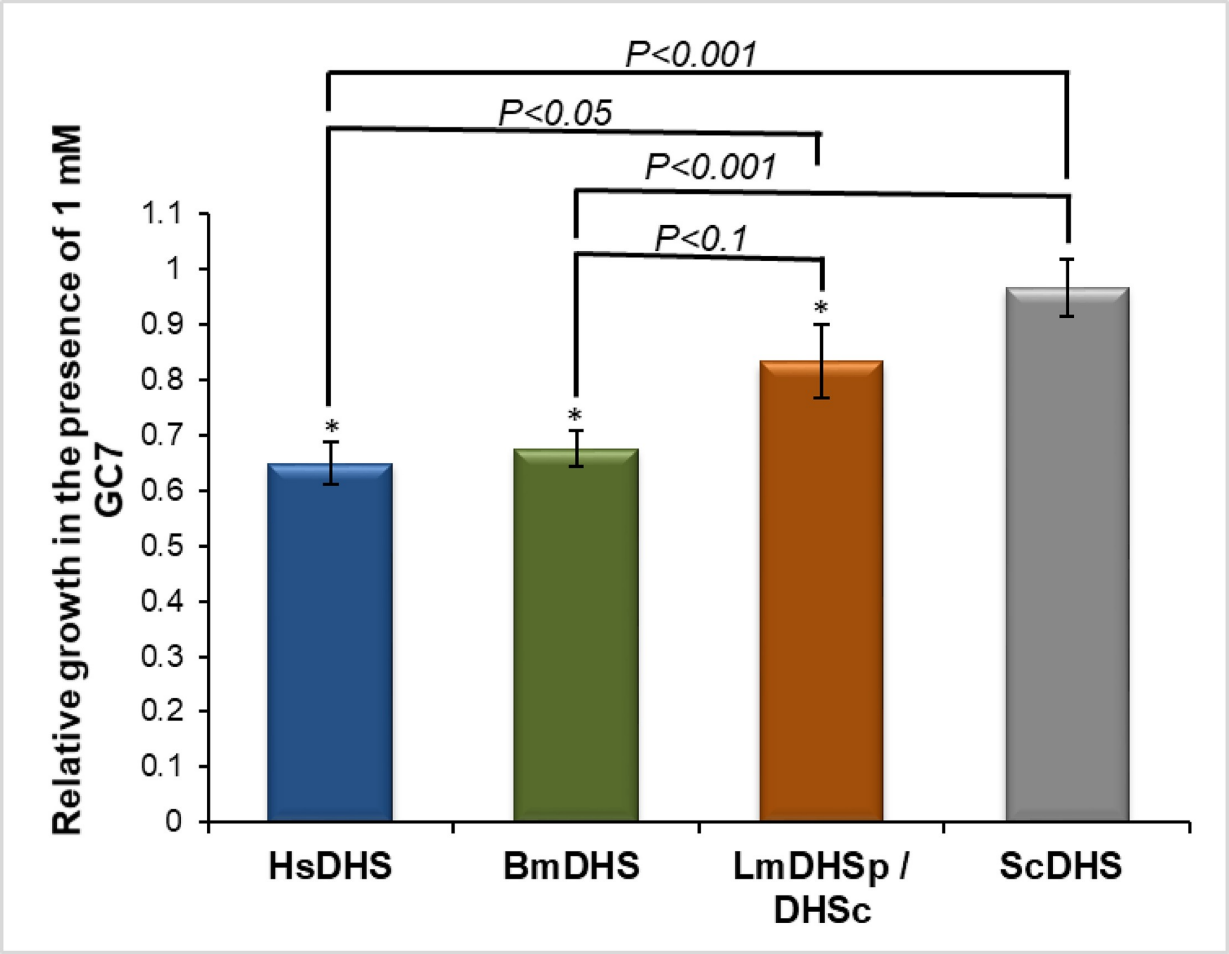
Yeast-based assay can detect DHS inhibitors. Bars show the relative growth score of *dys1Δ S*. *cerevisiae* strains complemented by the indicated DHS enzymes and the relative growth score of the wild type and isogenic strain VZL1462 (ScDHS—Table 2) in the presence of 1 mM GC7. A single star (*) indicate statistically significant differences (Student’s t-test p < 0.05) between the growth score of individual strains in the presence or absence of GC7.

To demonstrate this system can be used to identify compounds that inhibit DHS activity, we tested the effect of DHS inhibitor GC7 on the growth of *dys1Δ* mutants expressing different deoxyhypusine synthase homologs. GC7 impact in cell growth was more pronounced in *dys1Δ* cells ectopically expressing the human and the filarial worm DHS proteins (approximately 35% reduction in growth rate in the presence of 1 mM GC7) than on those complemented by LmDHSp/DHSc (approximately 15% reduction in growth rate in the presence of 1 mM GC7) (Fig 7 and S11 Fig). In addition, the ScDHS strain (wild-type) was less sensitive to GC7 in comparison to the heterologous strains. Taking into account that these strains have isogenic genetic background, the differences in sensitivity to GC7 observed among strains complemented with DHS from different species must be linked to the differential ability of GC7 to inhibit these enzymes (Fig 7 and S11 Fig). Our results suggested that these yeast strains could be used to identify new small molecules active against DHS from the filarial worm and *L*. *major*.

To evaluate whether the slower apparent growth of DHS-complemented yeast strains after GC7 treatment was due to higher mortality of cells, we performed a viability assay with those strains using the methylene blue staining technique (S12 Fig). As shown in the S12 Fig, all DHS-complemented strains in both GC7 treated and untreated conditions presented high cellular viability (> 98.5%). Therefore, GC7 did not affect the viability of these cells, and the slower growth of DHS-complemented yeast strains after GC7 treatment was not due to higher mortality. Likewise, a recent study with human colorectal cancer cells HCT-116 has demonstrated that GC7 treatment did not affect the viability of these cells [84]. Also, another study showed that GC7 is well tolerated by RAW264.7 murine macrophage cell [85].

## Discussion

Functional mammalian DHS are homotetrameric enzymes and employ a “ball and chain” mechanism to regulate substrate access to the complex four catalytic sites [16,17]. Our crystal structure of BmDHS showed the worm enzyme adopts the same oligomeric state and has a similar architecture to the human enzyme, including the presence of a N-terminal “ball and chain” region that blocks access to the active sites within the homotetramer. This blockage of BmDHS active sites by the conserved “ball and chain” region might explain the absence of GC7 in BmDHS crystals, despite this inhibitor being present at molar excess concentrations in the crystallization solution. A similar observation was made for HsDHS crystals [16]. Unfortunately, our attempts to obtain BmDHS in the open conformation using crystallization cocktails at different pHs, as shown for HsDHS [17], were not successful. An alternative strategy to obtain co-crystals of BmDHS bound to ligands might be to use truncated versions of the worm protein lacking the conserved α1 “ball” helix (residues 1–16). Nevertheless, possible co-structures of truncated DHS must be interpreted carefully, as HsDHS lacking the first 52 N-terminal residues, which include the whole ball and chain motif, behaves as a dimer in solution and are catalytically inactive [44].

By contrast, *T*. *brucei* DHS is a heterotetrameric protein, consisting of two DHSp/DHSc heterodimers [19]. Our homology model of the DHS paralogs from *Leishmania*, and our results in yeast (see above), suggested *L*. *major* DHSp/DHSc proteins adopt the same heterotetrameric architecture, with two catalytically active and two catalytically dead sites, as observed for the *T*. *brucei* proteins [19]. Kinetoplastid DHSp/DHSc lack the conserved N-terminal “ball and chain” region of their homotetrameric counterparts, implying that the control of substrate access to the active site, if present, must occur via a different mechanism in heterotetrameric DHS. Nevertheless, the size and location of four unique inserts in LmDHSc (between α3-β1, α5-β2, β4-β5, and α11-α12) suggest these might play a role in controlling access to the heterotetramer catalytically active and dead sites.

Most DHS inhibitors are polyamine analogues of spermidine, the enzyme natural substrate [86]. These compounds are well-suited to exploit multiple contact points along the enzyme funnel-shaped spermidine-binding site. As seen in the BmDHS crystal structure reported here, the high level of sequence and structural conservation between the human and worm enzymes might make it difficult to develop worm-selective DHS inhibitors. On the other hand, as suggested by our homology model, first-shell residues lining the spermidine-binding sites of the leishmania and human enzymes are quite different. Thus, it might be feasible to develop leishmania-selective DHS inhibitors with compounds targeting the enzyme active site. However, care should be taken when developing spermidine-based DHS inhibitors for parasite enzymes, as these are likely to interfere with spermidine biosynthesis and metabolism and cause undesired off-target effects in the human host [87].

Recently, Tanaka and colleagues identified and developed allosteric HsDHS inhibitors unrelated to spermidine. The most promising compound of this series (**11g** in reference [20]) caused the unfolding and relocation of α13 in HsDHS, thus creating a new allosteric pocket and, in the process, preventing binding of the NAD^+^ cofactor to the enzyme. These observations strengthen the notion that DHS enzymes are indeed druggable targets. Additionally, targeting allosteric sites is a validated strategy to achieve homologue selectivity [88].

The establishment of an easily-miniaturized, low-cost biochemical assay for BmDHS opens up the possibility of undertaking high-throughput screening (HTS) campaigns to find new ligands for the worm enzyme. Our assay is based on the accumulation of NADH produced during the partial DHS reaction in the absence of eIF5A. Using this assay, we found an IC_50_ value of 28 nM for GC7 against BmDHS activity. This value is in excellent agreement with the ones found for the mammalian enzymes in the presence of excess NAD^+^ cofactor and of both substrates required for the complete DHS reaction—spermidine and eIF5A (reported IC_50_ values ranging from 17–50 nM) [20,75,86]. We expected to find similar IC_50_ values for both complete and partial DHS reactions, as GC7 competes with spermidine, the substrate for the first step in the DHS reaction. The enzymatic assay used here was robust and sensitive over the μM to pM inhibitor concentration range employed, using a small amount of enzyme (in the low nanomolar range). For HTS campaigns, which generally use a single compound concentration, we expect it might be possible to further reduce the amount of enzyme, substrate and cofactor without compromising the sensitivity of the assay. It is also important to note that the enzymatic assay employed here would only be able to identify inhibitors of the partial DHS reaction.

Our biochemical data further suggested that BmDHS has a similar reaction mechanism to that observed for the mammalian enzymes, in which the NAD^+^-dependent dehydrogenation of spermidine to form dehydrospermidine is the rate-limiting step, with binding of the enzyme to both spermidine and NAD^+^ cofactor happening at much faster rates [89]. The same appears to be true for BmDHS, as we observe a linear dependence of NADH production with enzyme concentration, even when similar concentrations of enzyme and cofactor were used.

The yeast-based system developed here can also be used to screen small molecule inhibitors of the *Leishmania* DHS complex, which we could not produce recombinantly in *E*. *coli*. Rescue of yeast *dys1Δ* mutants required expression of both LmDHSp and LmDHSc proteins, thus suggesting that in *L*. *major*, as in *T*. *brucei*, the two DHS paralogs are required to form a functional DHS complex. Likewise, expression of BmDHS and HsDHS could also rescue *dys1Δ* mutants. We confirmed that the endogenous SceIF5A was hypusinated in *S*. *cerevisiae dys1Δ* strains expressing heterologous DHS proteins. This observation strongly suggested that, despite differences in sequence, structure and, possibly, regulatory mechanisms, LmDHSp/DHSc and BmDHS recognized and modified *S*. *cerevisiae* eIF5A. Moreover, GC7 negatively impacted the growth rate of DHS-complemented *dys1Δ* yeast strains, in a manner consistent with the inhibitor *in vitro* activity against the corresponding enzymes. GC7 is approximately 100-fold more potent against the worm and human enzymes than to the heterotetrameric TbDHS (reported IC_50_ value of 1.5 μM) [15]. We expect GC7 would also show decreased activity towards *L*. *major* DHS paralogs, given its similarity to the *T*. *brucei* proteins. Nevertheless, it is important to note that we had to use a large amount of GC7 (1 mM final concentration) to observe an effect in yeast growth rate, most likely due to the compound poor permeability into yeast cells.

In conclusion, here we developed the basis for target-based drug discovery programs to support hit identification and optimization for two high-value targets—the DHS enzymes from the filarial worm nematode, *B*. *malayi*, and *L*. *major*.

## Supporting information

S1 Fig Determination of the best pH for the enzymatic assay with BmDHS. Reaction progression curves showing the effect of different pH (9.6, 9.2, 8.8, 8.4 and 8.0) in the activity of BmDHS. The reaction was composed by 3.6 μM BmDHS, 17 μM spermidine and 19 μM NAD^+^ [47]. The individual points represent the mean ± standard error of experimental duplicates.
(TIF)

S2 Fig SDS-PAGE analysis of eluted fractions obtained from small-scale test expression in BL21(DE3)-R3-pRARE2 strain for BmDHS constructs. M: molecular weight marker (Precision Plus Protein Unstained Protein Standards, BioRad, cat no. 161–0363). Samples are identified according to their construct IDs (S2 Table). Expected sizes (in Da): BmDHS-cb001 = 43,548.6, BmDHS-cb002 = 43,319.4, BmDHS-cb003 = 43,045.1, and BmDHS-cb004 = 42,769.7.
(TIF)

S3 Fig Recombinant BmDHS can be purified and is a tetramer in solution. (A) SDS-PAGE analysis of recombinant BmDHS purification. IMAC fractions: total lysate (LT), soluble fraction (FS), Ni-NTA flow-through (FT), Ni-NTA eluate with 300 mM imidazole (E). Following TEV protease treatment (TEV), the mixture was applied to a second IMAC step using Ni^2+^-charged Ni-NTA resin. IMAC fractions: flow through (R1), wash with 30 mM imidazole (R2) and elution with 300 mM imidazole (R3). M: molecular weight marker (Precision Plus Protein Unstained Protein Standards, BioRad, cat no. 161–0363). (B) Chromatogram of fraction R1 separated by gel filtration chromatography (GF). (C) SDS-PAGE analysis of gel filtration samples in panel B. (D) Graph showing the apparent partition coefficients for protein standard (in black font) and BmDHS (in red font) following analytical gel filtration chromatography. (E) Deconvoluted spectrum for recombinant BmDHS subjected to mass spectrometry analysis.
(TIF)

S4 Fig SDS-PAGE analysis of total lysate (TL) and eluted (E) fractions obtained from small-scale test expression in BL21(DE3)-R3-pRARE2 strain for different versions of LmDHSc and LmDHSp constructs and LmDHSp/DHSc construct. M: molecular weight marker (PageRuler Prestained Protein Ladder, ThermoFisher Scientific, cat n^o^. 26616). Samples are identified according to their construct IDs (S2 Table). Expected sizes (in Da): LmDHSc-cb001 = 66,890.7, LmDHSc-cb002 = 64,062.6, LmDHSc-cb003 = 64,833.4, LmDHSc-cb004 = 65,913.6, LmDHSp-cb001 = 43,377.7, LmDHSp-cb002 = 42,613.8, LmDHSp-cb003 = 42,074.2, and LmDHSp-cb004 = 40,457.4. Co-expression of LmDHSc-cb001 and LmDHSp-cb001 was performed in pET-DUET1 (Table 1).
(TIF)

S5 Fig Predicted GC7 interactions in BmDHS crystal structure. (A) Protomer A1 is shown as a white surface, with residues within a 4 Å radius of the NAD^+^ cofactor (spheres) shown as blue sticks and highlighted by pale blue surface. Residues in protomer A2 within a 4 Å radius of the NAD^+^ cofactor bound to protomer A1 are shown as pink sticks. The NAD^+^ cofactor bound to protomer A2 is also shown as pink sticks. Protomers B1 (yellow) and B2 (green) are shown as cartoon. (B, C) Close view showing catalytically-important residues within BmDHS active site. GC7 (yellow stick) was docked following the superposition of the crystal structure of BmDHS onto the crystal structure of GC7-bound HsDHS (PDB ID 1RQD) using Pymol (Schrödinger, Inc).
(TIF)

S6 Fig Structure-based sequence alignment of various eIF5A. The protein stretches showing the most conserved sequences are depicted in blue boxes. The residues written in light red are similar and the ones written in white and boxed in red are identical residues. The secondary structure (α-helices and β-sheets), and the numbering shown in the top line are for *Saccharomyces cerevisiae* eIF5A1 (PDB: 3ER0). UniProt IDs for protein sequences used in the alignment were: *Pyrococcus horikoshii* Ph-eIF5A - O50089, Lm-eIF5A - Q4QA21, Tb-eIF5A - Q387H6, *Entamoeba dispar* Ed-eIF5A - B0E9L6, Bm-eIF5A - A0A0I9R327, Sc-EiF5A1—P23301, SceIF5A2—P19211, Danio rerio Dr-eIF5A1—Q6NX89, Dr-eIF5A2—Q7ZUP4, Hs-eIF5A1—P63241, Hs-EIF5A2—Q9GZV4.
(TIF)

S7 Fig Local quality estimate of residues in the homology model of *L*. *major* DHS heterotetramer. Graphical representation of the predicted local similarity (Y-axis) between individual residues (X-axis) in the final SWISS-MODEL LmDHSp/DHSc homology model and the TbDHSp/DHSc target structure (PDB ID 6DFT) [19]. Local quality estimates are shown for LmDHSp (left panel) and LmDHSc (right panel) protomers. The threshold for poor- and high-quality local similarity regions is 0.6 (indicated by a black dashed line). The arrowhead indicates the position of the catalytic lysine residue in LmDHS (Lys535).
(TIF)

S8 Fig Homology model of *L*. *major* DHS heterotetramer. (A) Cartoon representation of the LmDHSp/DHSc heterotetramer. Individual LmDHSc and LmDHSp protomers were colored differently based on secondary structure (LmDHSc—helices: red, sheets: yellow, loops: green; and LmDHSp—helices: cyan, sheets: red, and coils: magenta). (B, C) Individual LmDHSp (B) and LmDHSc (C) protomers superposed onto the equivalent proteins from the crystal structure of the ternary complex formed by NAD^+^-TbDHSp/DHSc and used as template (PDB ID: 6DFT) [19] for modelling. Coloring scheme for LmDHS as in panel A, *T*. *brucei* proteins are shown in gray. The NAD^+^ cofactor is shown in sphere representation. In panel C, the “ball” α-helix from HsDHS is shown in blue cartoon as it would block entrance to one of the two active sites in a homodimer for the human enzyme.
(TIF)

S9 Fig Predicted NAD^+^ interactions in LmDHSp/DHSc structural model. (A) Overlay of NAD^+^-binding sites in HsDHS (PDB ID 1RQD) and the corresponding region in the LmDHSp/DHSc homology model. (B, C) LmDHS residues interacting with NAD^+^ in the catalytic site (panel B) and in the dead site (panel C). (D) Overlay of NAD^+^-binding sites in TbDHSp/DHSc (PDB ID 6DFT) and the corresponding region in the LmDHSp/DHSc homology model. In panels B-D colors and notations are: blue—LmDHS; green—HsDHS, and yellow—TbDHS; La: LmDHSc; Lb: LmDHSp; h: HsDHS; Ta: TbDHSc; and Tb: TbDHSp.
(TIF)

S10 Fig Reaction progress curves of enzymatic assays with BmDHS. (A) Reaction progress curves in the presence of increasing concentrations of the cofactor NAD^+^ (0.195 to 400 μM), 100nM BmDHS and a fixed excess concentration of the substrate spermidine (0.56 μM). (B) Reaction progress curves in the presence of increasing concentrations of the substrate spermidine (0.0005 to 1 μM), 100nM BmDHS and a fixed excess of the cofactor NAD^+^ (132 μM). The individual points represent the mean ± standard error of experimental duplicates. Shown are representative curves from a single experiment. All experiments were performed at least twice.
(TIF)

S11 Fig Growth analysis of yeast deoxyhypusine synthase deleted strain complemented by orthologue enzymes. Comparison of inhibition caused by the GC7 compound in yeast strains complemented with DHS from *Homo sapiens* (A); two isoforms from *Leishmania major* (B); and *Brugia malayi* (C).
(TIF)

S12 Fig Cell viability estimated with methylene blue dye. ScDHS and DHS-complemented yeast strains after incubation with 1 mM GC7 or with the vehicle (0 mM GC7) were labeled with methylene blue for 5 min at room temperature. ScDHS was also treated with 10 mM H_2_O_2_ as a positive control of decrease of yeast cell viability (control). At least 320 cells were examined under a light microscope in each condition. The results are presented as the mean of the percentage of viable cells ± standard deviation from two biological replicates. The difference in viabilities was deemed statistically significant by the Student's t-test comparing cells grown in the in the presence or absence of GC7; *** p<0.001 in comparison to other conditions.
(TIF)

S1 File Multiple alignment of the sequence generated by DNA sequencing of the BmDHS, LmDHSp and LmDHSc full-length constructs. (ZIP)

S1 Table Oligonucleotide sequences. (DOCX)

S2 Table Detailed information for BmDHS and LmDHS constructs. (DOCX)

S3 Table Comparison of active site amino acids between BmDHS and its counterparts in *H*. *sapiens* (HsDHS), *T*. *brucei* (TbDHSp/DHSc) and *L*. *major* (LmDHSp/DHSc). (DOCX)
