# Supplementary figures and images for: Structural features and development of an assay platform of the parasite target deoxyhypusine synthase of Brugia malayi and Leishmania major
Source: PLoS Negl Trop Dis. 2020 Oct 12;14(10):e0008762. doi: 10.1371/journal.pntd.0008762 (PMC7581365; doi:10.1371/journal.pntd.0008762)

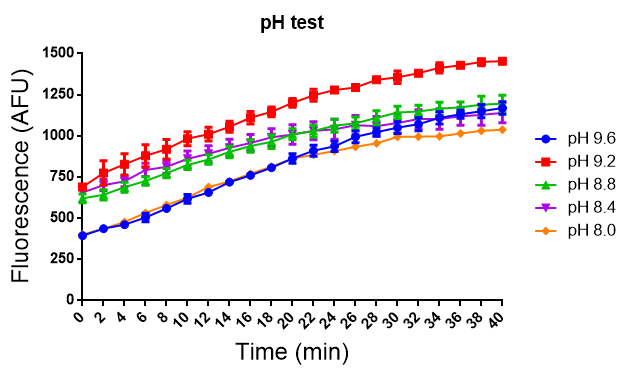

Supplement: S1 Fig — The reaction was composed by 3.6 μM BmDHS, 17 μM spermidine and 19 μM NAD+ [47]. The individual points represent the mean ± standard error of experimental duplicates. (TIF) [file pntd.0008762.s001.tif]

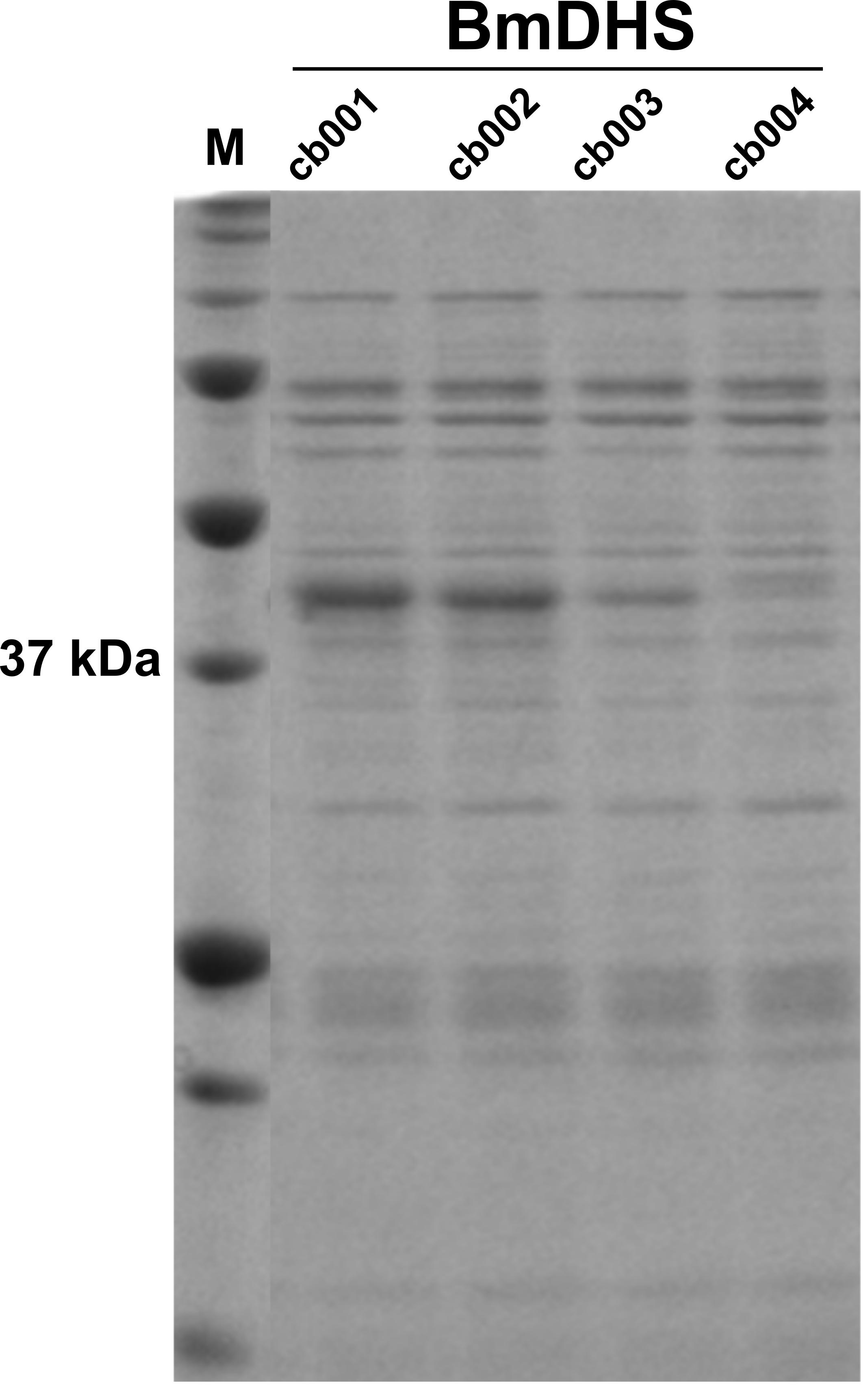

Supplement: S2 Fig — M: molecular weight marker (Precision Plus Protein Unstained Protein Standards, BioRad, cat no. 161–0363). Samples are identified according to their construct IDs (S2 Table). Expected sizes (in Da): BmDHS-cb001 = 43,548.6, BmDHS-cb002 = 43,319.4, BmDHS-cb003 = 43,045.1, and BmDHS-cb004 = 42,769.7. (TIF) [file pntd.0008762.s002.tif]

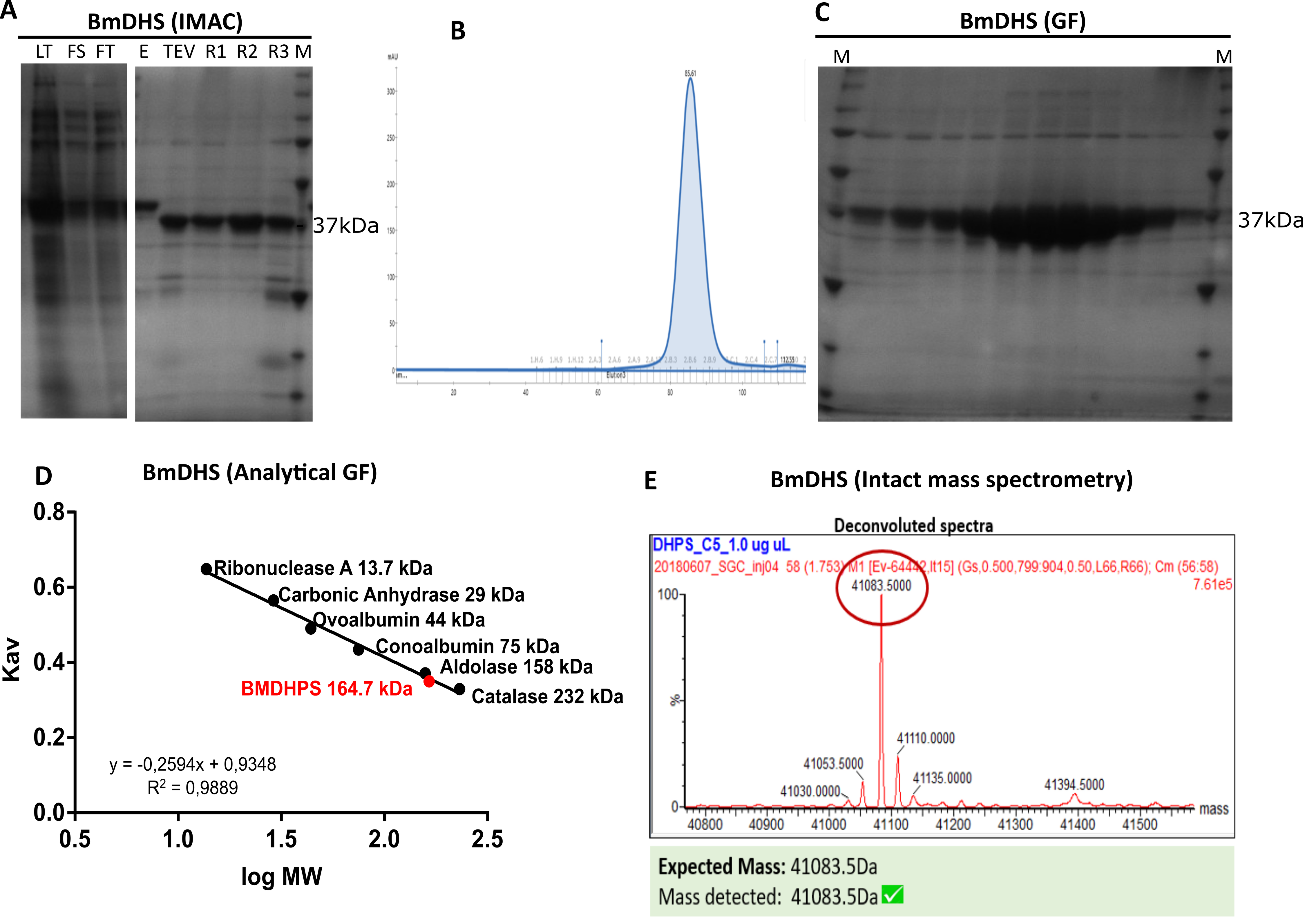

Supplement: S3 Fig — (A) SDS-PAGE analysis of recombinant BmDHS purification. IMAC fractions: total lysate (LT), soluble fraction (FS), Ni-NTA flow-through (FT), Ni-NTA eluate with 300 mM imidazole (E). Following TEV protease treatment (TEV), the mixture was applied to a second IMAC step using Ni2+-charged Ni-NTA resin. IMAC fractions: flow through (R1), wash with 30 mM imidazole (R2) and elution with 300 mM imidazole (R3). M: molecular weight marker (Precision Plus Protein Unstained Protein Standards, BioRad, cat no. 161–0363). (B) Chromatogram of fraction R1 separated by gel filtration chromatography (GF). (C) SDS-PAGE analysis of gel filtration samples in panel B. (D) Graph showing the apparent partition coefficients for protein standard (in black font) and BmDHS (in red font) following analytical gel filtration chromatography. (E) Deconvoluted spectrum for recombinant BmDHS subjected to mass spectrometry analysis. (TIF) [file pntd.0008762.s003.tif]

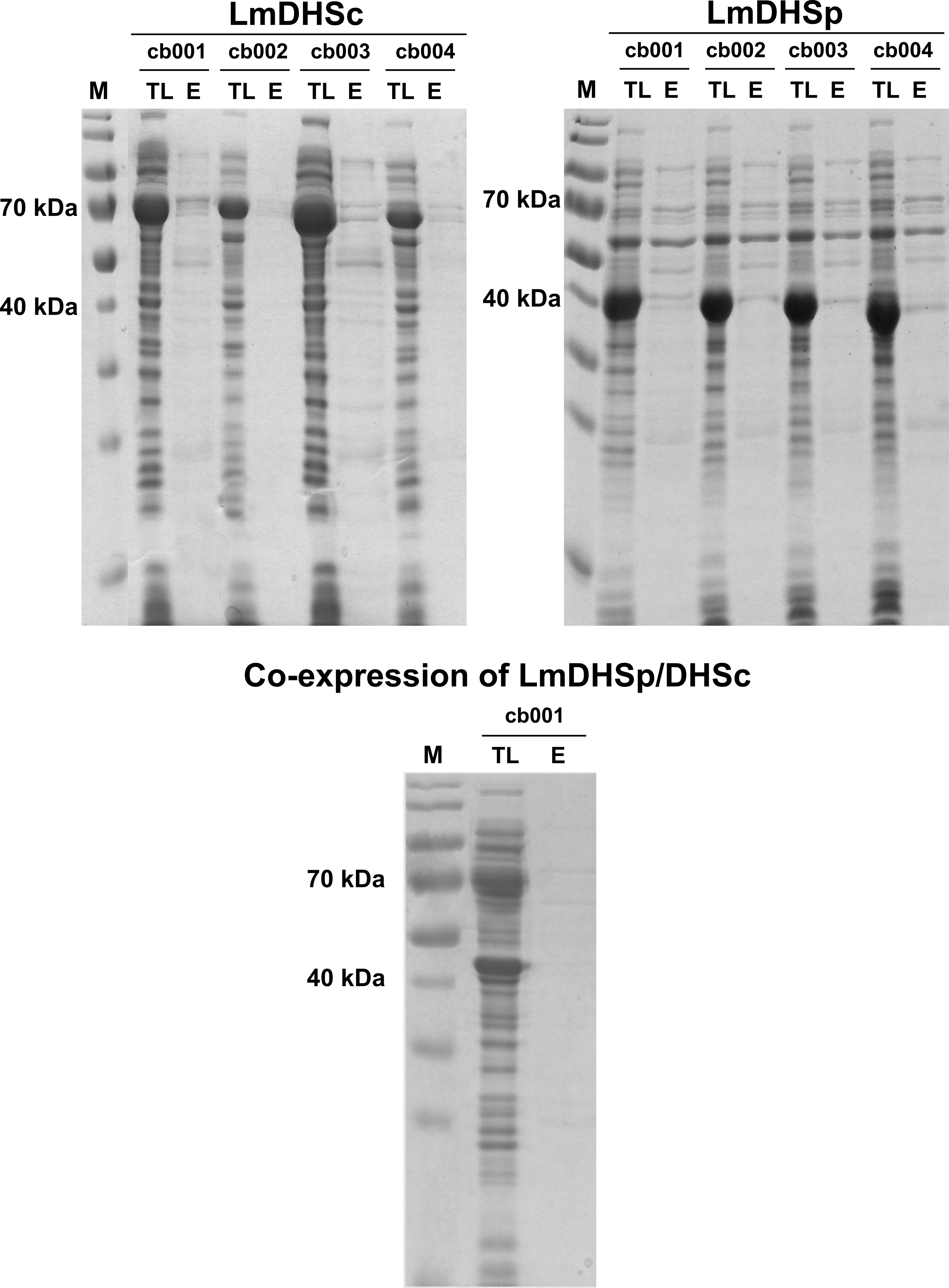

Supplement: S4 Fig — M: molecular weight marker (PageRuler Prestained Protein Ladder, ThermoFisher Scientific, cat no. 26616). Samples are identified according to their construct IDs (S2 Table). Expected sizes (in Da): LmDHSc-cb001 = 66,890.7, LmDHSc-cb002 = 64,062.6, LmDHSc-cb003 = 64,833.4, LmDHSc-cb004 = 65,913.6, LmDHSp-cb001 = 43,377.7, LmDHSp-cb002 = 42,613.8, LmDHSp-cb003 = 42,074.2, and LmDHSp-cb004 = 40,457.4. Co-expression of LmDHSc-cb001 and LmDHSp-cb001 was performed in pET-DUET1 (Table 1). (TIF) [file pntd.0008762.s004.tif]

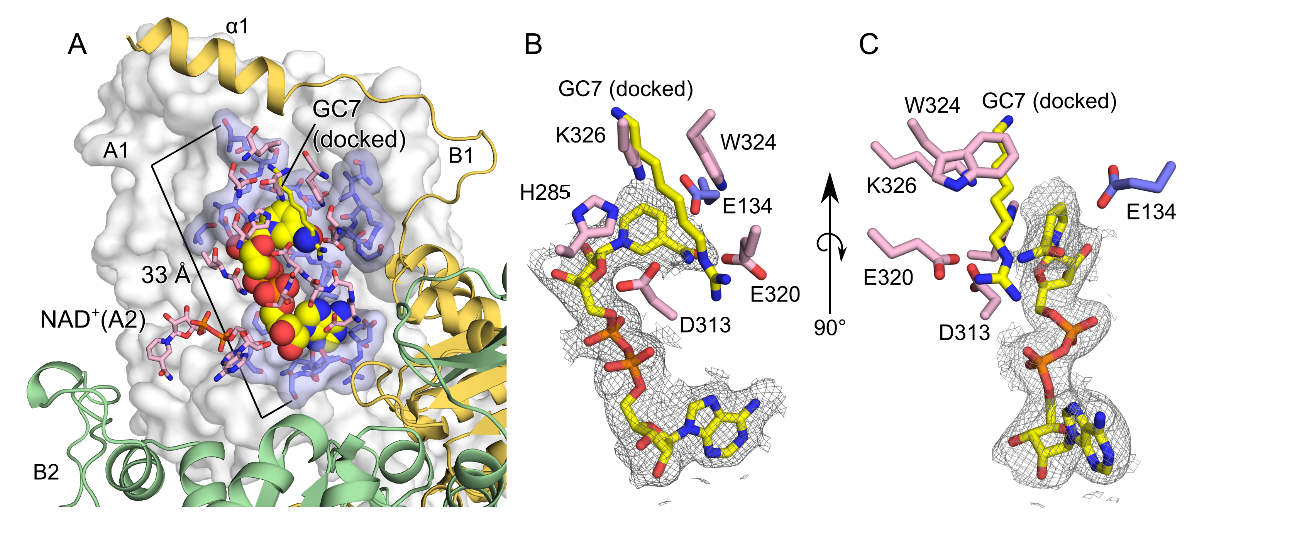

Supplement: S5 Fig — (A) Protomer A1 is shown as a white surface, with residues within a 4 Å radius of the NAD+ cofactor (spheres) shown as blue sticks and highlighted by pale blue surface. Residues in protomer A2 within a 4 Å radius of the NAD+ cofactor bound to protomer A1 are shown as pink sticks. The NAD+ cofactor bound to protomer A2 is also shown as pink sticks. Protomers B1 (yellow) and B2 (green) are shown as cartoon. (B, C) Close view showing catalytically-important residues within BmDHS active site. GC7 (yellow stick) was docked following the superposition of the crystal structure of BmDHS onto the crystal structure of GC7-bound HsDHS (PDB ID 1RQD) using Pymol (Schrödinger, Inc). (TIF) [file pntd.0008762.s005.tif]

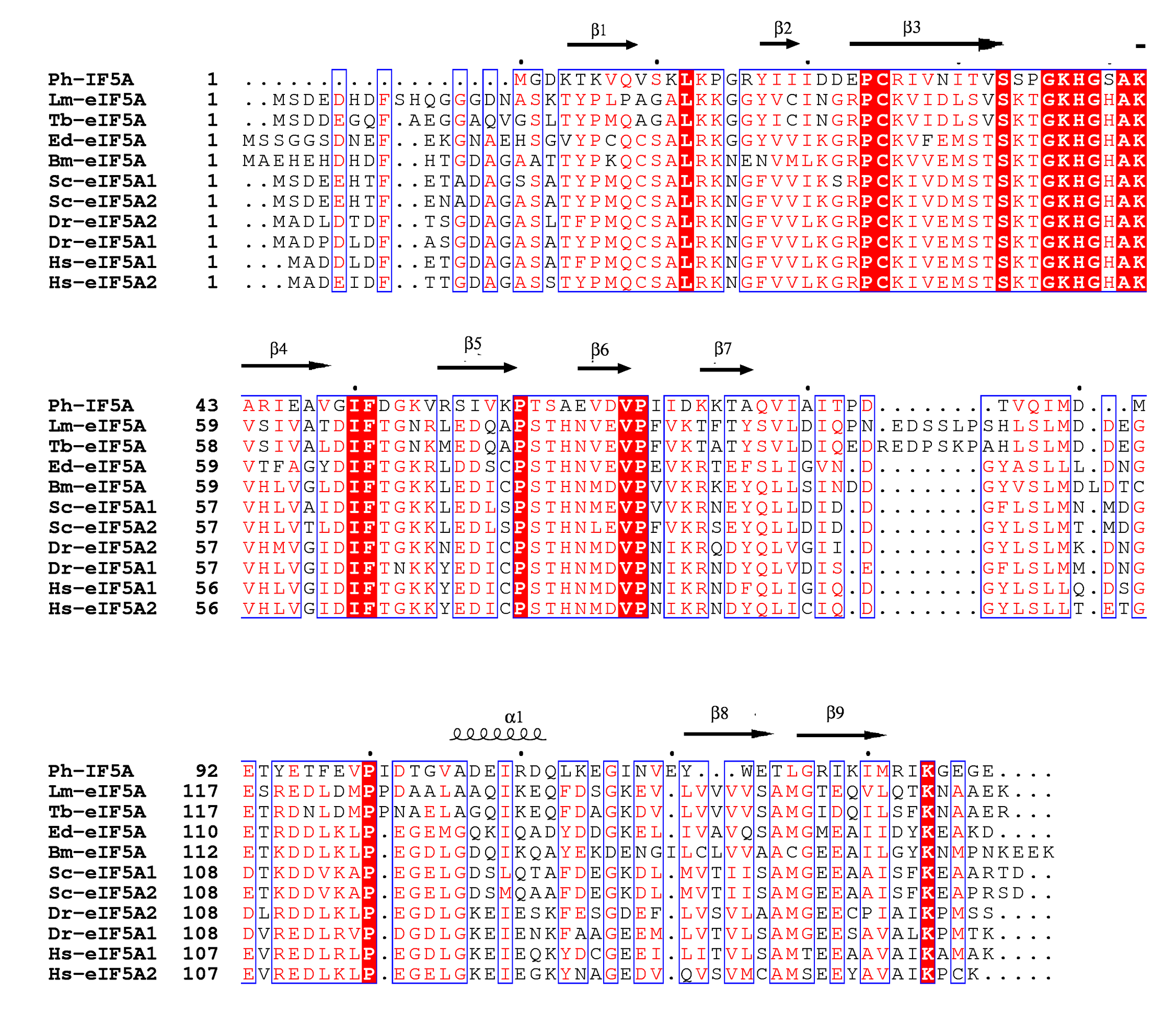

Supplement: S6 Fig — The protein stretches showing the most conserved sequences are depicted in blue boxes. The residues written in light red are similar and the ones written in white and boxed in red are identical residues. The secondary structure (α-helices and β-sheets), and the numbering shown in the top line are for Saccharomyces cerevisiae eIF5A1 (PDB: 3ER0). UniProt IDs for protein sequences used in the alignment were: Pyrococcus horikoshii Ph-eIF5A - O50089, Lm-eIF5A - Q4QA21, Tb-eIF5A - Q387H6, Entamoeba dispar Ed-eIF5A - B0E9L6, Bm-eIF5A - A0A0I9R327, Sc-EiF5A1—P23301, SceIF5A2—P19211, Danio rerio Dr-eIF5A1—Q6NX89, Dr-eIF5A2—Q7ZUP4, Hs-eIF5A1—P63241, Hs-EIF5A2—Q9GZV4. (TIF) [file pntd.0008762.s006.tif]

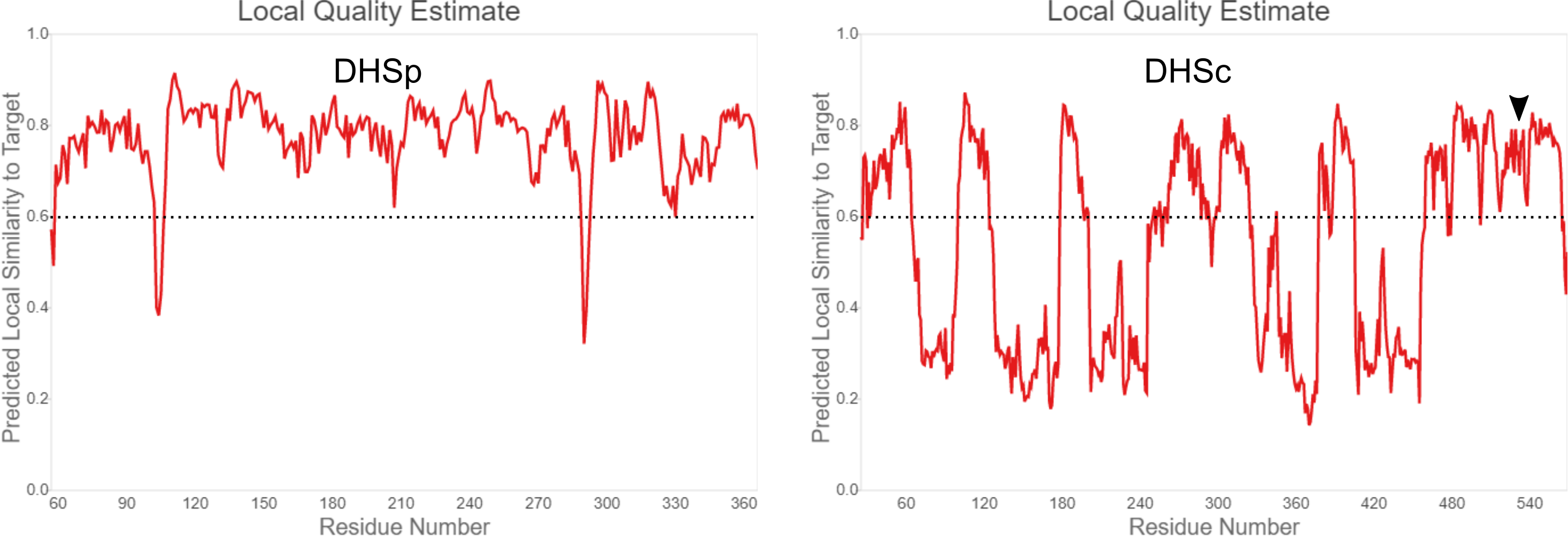

Supplement: S7 Fig — Graphical representation of the predicted local similarity (Y-axis) between individual residues (X-axis) in the final SWISS-MODEL LmDHSp/DHSc homology model and the TbDHSp/DHSc target structure (PDB ID 6DFT) [19]. Local quality estimates are shown for LmDHSp (left panel) and LmDHSc (right panel) protomers. The threshold for poor- and high-quality local similarity regions is 0.6 (indicated by a black dashed line). The arrowhead indicates the position of the catalytic lysine residue in LmDHS (Lys535). (TIF) [file pntd.0008762.s007.tif]

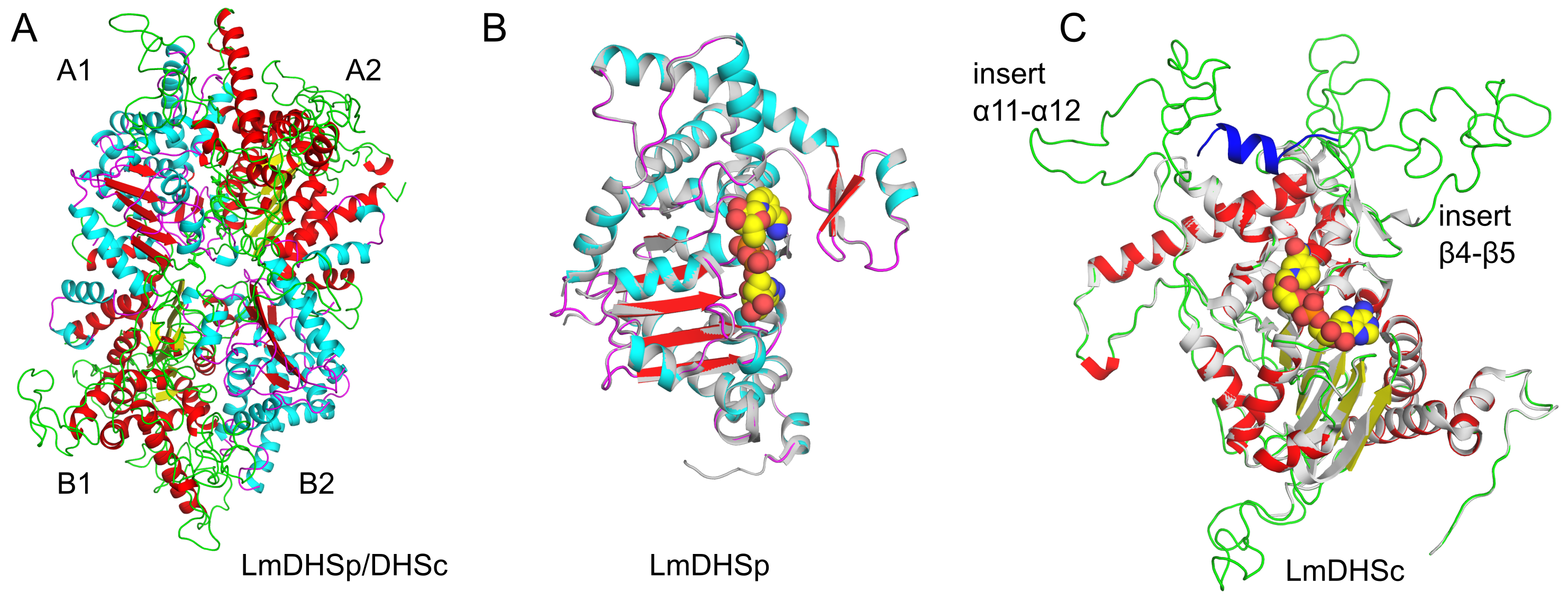

Supplement: S8 Fig — (A) Cartoon representation of the LmDHSp/DHSc heterotetramer. Individual LmDHSc and LmDHSp protomers were colored differently based on secondary structure (LmDHSc—helices: red, sheets: yellow, loops: green; and LmDHSp—helices: cyan, sheets: red, and coils: magenta). (B, C) Individual LmDHSp (B) and LmDHSc (C) protomers superposed onto the equivalent proteins from the crystal structure of the ternary complex formed by NAD+-TbDHSp/DHSc and used as template (PDB ID: 6DFT) [19] for modelling. Coloring scheme for LmDHS as in panel A, T. brucei proteins are shown in gray. The NAD+ cofactor is shown in sphere representation. In panel C, the “ball” α-helix from HsDHS is shown in blue cartoon as it would block entrance to one of the two active sites in a homodimer for the human enzyme. (TIF) [file pntd.0008762.s008.tif]

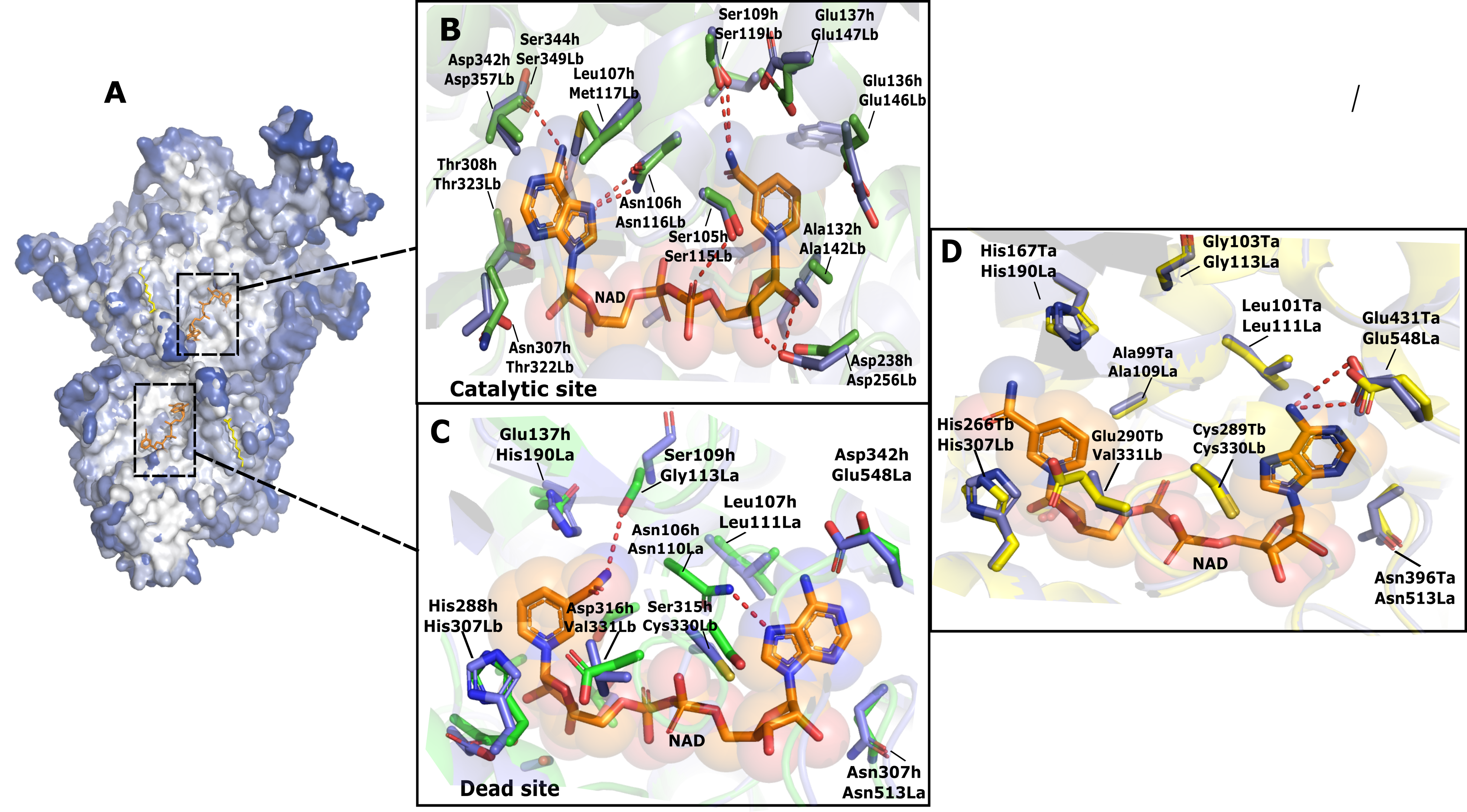

Supplement: S9 Fig — (A) Overlay of NAD+-binding sites in HsDHS (PDB ID 1RQD) and the corresponding region in the LmDHSp/DHSc homology model. (B, C) LmDHS residues interacting with NAD+ in the catalytic site (panel B) and in the dead site (panel C). (D) Overlay of NAD+-binding sites in TbDHSp/DHSc (PDB ID 6DFT) and the corresponding region in the LmDHSp/DHSc homology model. In panels B-D colors and notations are: blue—LmDHS; green—HsDHS, and yellow—TbDHS; La: LmDHSc; Lb: LmDHSp; h: HsDHS; Ta: TbDHSc; and Tb: TbDHSp. (TIF) [file pntd.0008762.s009.tif]

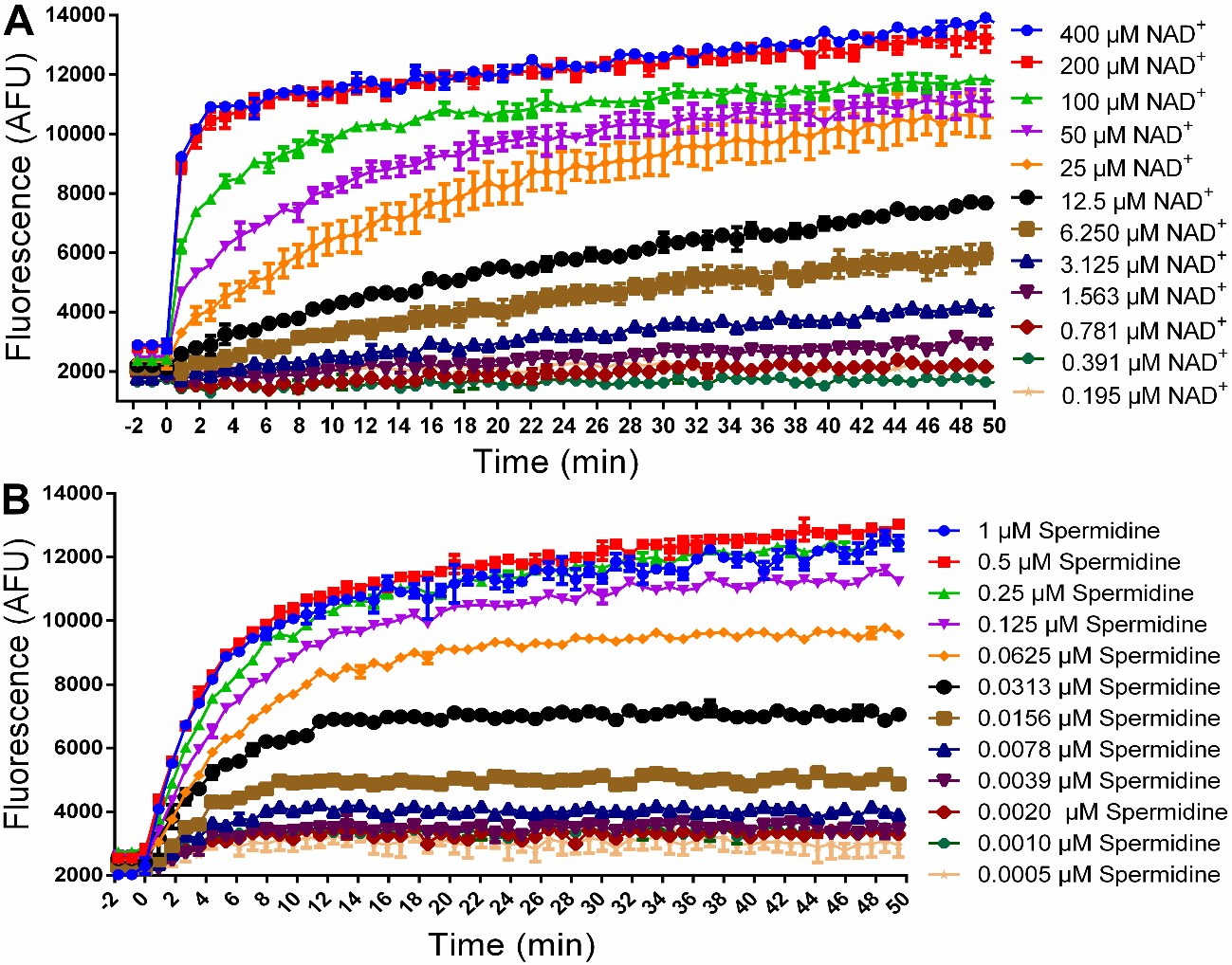

Supplement: S10 Fig — (A) Reaction progress curves in the presence of increasing concentrations of the cofactor NAD+ (0.195 to 400 μM), 100nM BmDHS and a fixed excess concentration of the substrate spermidine (0.56 μM). (B) Reaction progress curves in the presence of increasing concentrations of the substrate spermidine (0.0005 to 1 μM), 100nM BmDHS and a fixed excess of the cofactor NAD+ (132 μM). The individual points represent the mean ± standard error of experimental duplicates. Shown are representative curves from a single experiment. All experiments were performed at least twice. (TIF) [file pntd.0008762.s010.tif]

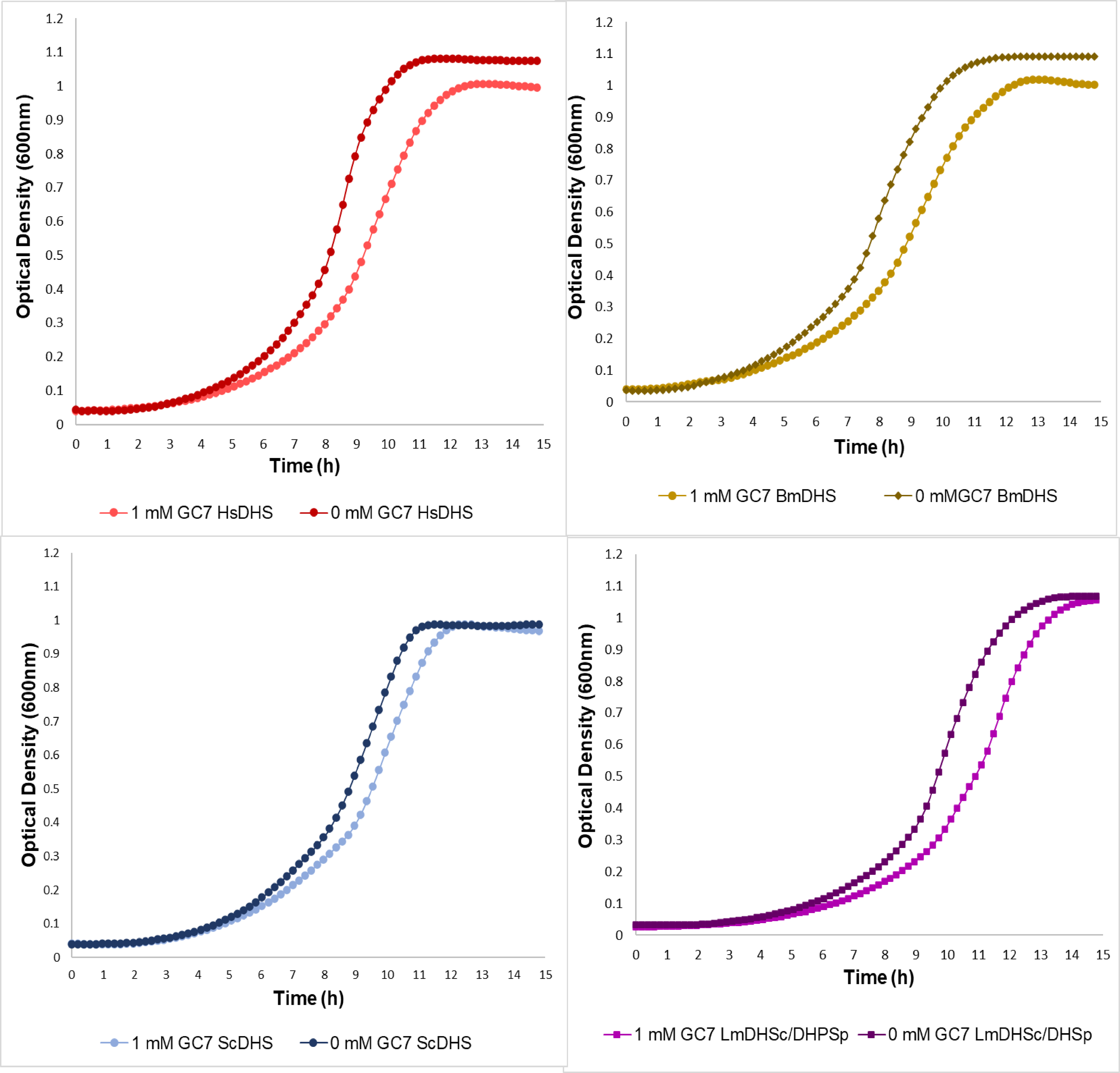

Supplement: S11 Fig — Comparison of inhibition caused by the GC7 compound in yeast strains complemented with DHS from Homo sapiens (A); two isoforms from Leishmania major (B); and Brugia malayi (C). (TIF) [file pntd.0008762.s011.tif]

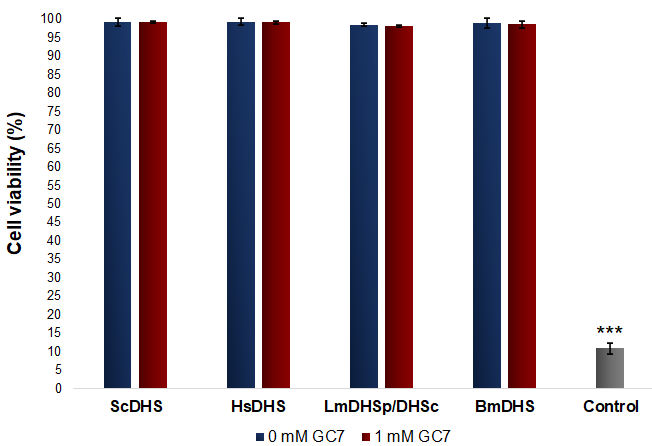

Supplement: S12 Fig — ScDHS and DHS-complemented yeast strains after incubation with 1 mM GC7 or with the vehicle (0 mM GC7) were labeled with methylene blue for 5 min at room temperature. ScDHS was also treated with 10 mM H2O2 as a positive control of decrease of yeast cell viability (control). At least 320 cells were examined under a light microscope in each condition. The results are presented as the mean of the percentage of viable cells ± standard deviation from two biological replicates. The difference in viabilities was deemed statistically significant by the Student's t-test comparing cells grown in the in the presence or absence of GC7; *** p<0.001 in comparison to other conditions. (TIF) [file pntd.0008762.s012.tif]
