# Supplementary material for: Structural features and development of an assay platform of the parasite target deoxyhypusine synthase of Brugia malayi and Leishmania major
Source: PLoS Negl Trop Dis. 2020 Oct 12;14(10):e0008762. doi: 10.1371/journal.pntd.0008762 (PMC7581365; doi:10.1371/journal.pntd.0008762)
Supplement: S1 File — (ZIP) [file pntd.0008762.s013.zip › Multiple alignment of DNA sequencing.docx]

**Multiple alignment of DNA sequencing– BmDHS-cb001:**

CLUSTAL O(1.2.4) multiple sequence alignment

BmDHS ------------------------------------------------------------ 0

cb001F CGACTTTTTGCTTACTTTAGATGAGATATACATATGCACCATCATCATCATCATTCTTCT 60

cb001R ------------------------------------------------------------ 0

BmDHS ---------------------------------------ATGGACAACGGTAACTGTAAA 21

cb001F GGTGTAGATCTGGGTACCGAGAACCTGTACTTCCAATCCATGGACAACGGTAACTGTAAA 120

cb001R ------------------------------------------------------------ 0

BmDHS TTCGATGTTCATATCGCCGAAATGTCCGTCTTGAAGAAATCTTCTACTATGCCAGCTGAT 81

cb001F TTCGATGTTCATATCGCCGAAATGTCCGTCTTGAAGAAATCTTCTACTATGCCAGCTGAT 180

cb001R ------------------------------------------------------------ 0

BmDHS TCCACCATTATTAAGGGTTACGATTTCAACGAAGGTATCAACTACGATGCCTTGTTGGAC 141

cb001F TCCACCATTATTAAGGGTTACGATTTCAACGAAGGTATCAACTACGATGCCTTGTTGGAC 240

cb001R ------------------------------------------------------------ 0

BmDHS CAATATATGTCTACTGGTTTTCAAGCCTCTCATTTCGCTCAAGCTGTTCAACAAATCAAC 201

cb001F CAATATATGTCTACTGGTTTTCAAGCCTCTCATTTCGCTCAAGCTGTTCAACAAATCAAC 300

cb001R ----------------------------------------------GTTTGGAATCCCAT 14

* ** * *

BmDHS ACCATGTTGACCATCAGAGAAGAACAATTCGAAGGTGATCATACTT------TGCCATAC 255

cb001F ACCATGTTGACCATCAGAGAAGAACAATTCGAAGGTGATCATACTT------TGCCATAC 354

cb001R T---------CCAGGAGGAAGACCACTTCCGAAAGTGATCATACTTGACATATCCCAAGA 65

*** ** * * **** ************ * ***

BmDHS CCAGAAGGTAAACAAAAAAGAGCTTGCACCATTTTCTTGGGTTACACCTCTAATTTGGTT 315

cb001F CCAGAAGGTAAACAAAAAAGAGCTTGCACCATTTTCTTGGGTTACACCTCTAATTTGGTT 414

cb001R AAGGGTAAACAAAAAAAGAGCCTTGGCACCATTTTCTGGGTTAACACCTCTAATTTGGGT 125

* ** **** ** * ************ ** * *************** *

BmDHS ACCTCTGGTGTTAGAGAAAACATCAGATACTTGGTTGAACACGATTTGGTTGATTGCATC 375

cb001F ACCTCTGGTGTTAGAGAAAACATCAGATACTTGGTTGAACACGATTTGGTTGATTGCATC 474

cb001R ACCTCTGGTGTTAGAGAAAACATCAGATACTTGGTTGAACACGAT-TGGTTGATTGCATC 184

********************************************* **************

BmDHS GTTACATCTGCTGGTGGTGTTGAAGAAGATTTGATTAAGTGTTTGGCCCCATCTTACTTG 435

cb001F GTTACATCTGCTGGTGGTGTTGAAGAAGATTTGATTAAGTGTTTGGCCCCATCTTACTTG 534

cb001R GTTACATCTGCTGGTGGTGTTGAAGAAGATTTGATTAAGTGTTTGGCCCCATCTTACTTG 244

************************************************************

BmDHS GGTGCTTTTGATTTGGATGGTAAGACCTTGAGACATAACGGTTTGAATAGAGCCGGTAAC 495

cb001F GGTGCTTTTGATTTGGATGGTAAGACCTTGAGACATAACGGTTTGAATAGAGCCGGTAAC 594

cb001R GGTGCTTTTGATTGGGATGGTAAGACCTTGAGACATAACGGTTTGAATAGAGCCGGTAAC 304

************* **********************************************

BmDHS ATTATCATCCCAAACAACAACTACTGCCAATTTGAAGATTGGTTGATGCCAATCTTGGAC 555

cb001F ATTATCATCCCAAACAACAACTACTGCCAATTTGAAGATTGGTTGATGCCAATCTTGGAC 654

cb001R ATTATCATCCCAAACAACAACTACTGCCAATTTGAAGATTGGTTGATGCCAATCTTGGAC 364

************************************************************

BmDHS TCTTGTGAATTGGAACAAAAGAACAACGATTTCTCTTGGACCCCATCCAAGTTGATTGAT 615

cb001F TCTTGTGAATTGGAACAAAAGAACAACGATTTCTCTTGGACCCCATCCAAGTTGATTGAT 714

cb001R TCTTGTGAATTGGAACAAAAGAACAACGATTTCTCTTGGACCCCATCCAAGTTGATTGAT 424

************************************************************

BmDHS AGATTGGGTGCTGAAATCAACGACAAGAGATCTATTTGTTACTGGGCCCATAGAAACAGA 675

cb001F AGATTGGGTGCTGAAATCAACGACAAGAGATCTATTTGTTACTGGGCCCATAGAAACAGA 774

cb001R AGATTGGGTGCTGAAATCAACGACAAGAGATCTATTTGTTACTGGGCCCATAGAAACAGA 484

************************************************************

BmDHS ATCCCAGTTT-TTTCACCAGCTTTGACCGATGGTTCTATTGGTGATATGTTGTACTTCCA 734

cb001F ATCCCAGTTTTTTTCACCAGCTTTGACCGATGGTTCTATTGGTGATATGTTGTACTTCCA 834

cb001R ATCCCAGTTT-TTTCACCAGCTTTGACCGATGGTTCTATTGGTGATATGTTGTACTTCCA 543

********** *************************************************

BmDHS CTCTTTCAGAAACGG-TGGTATCAAGTTGGATATCGTCGAAGATTTGAGACACATTAACA 793

cb001F CTCTTTCAGAAACCGGTGGTATCAAGTTGGATATCGTCGAAGAATTTGAAGACAACATTA 894

cb001R CTCTTTCAGAAACGG-TGGTATCAAGTTGGATATCGTCGAAGATTTGAGACACATTAACA 602

************* * *************************** ** * *** * *

BmDHS CTATGGCTGTCAGATCTAACAGAACCGGTGTTATTTTGTTGGGTGGTGGTGTAATGAAGC 853

cb001F AACACCTA---------------------------------------------------- 902

cb001R CTATGGCTGTCAGATCTAACAGAACCGGTGTTATTTTGTTGGGTGGTGGTGTAATGAAGC 662

BmDHS ACCATATTAACAATGCTAACTTGATGAGAAACGGTTCCGATTACGCTGTTTACGTTAATA 913

cb001F ------------------------------------------------------------ 902

cb001R ACCATATTAACAATGCTAACTTGATGAGAAACGGTTCCGATTACGCTGTTTACGTTAATA 722

BmDHS CCGGTCAAGAATTTGATGGTTCTGATTCTGGTGCTAGACCTGATGAAGCTGTTTCTTGGG 973

cb001F ------------------------------------------------------------ 902

cb001R CCGGTCAAGAATTTGATGGTTCTGATTCTGGTGCTAGACCTGATGAAGCTGTTTCTTGGG 782

BmDHS GTAAAGTTAGATCAGATTGCAGACCAGTTAAGATCTATGCTGATGCTACTTTGGTTTTCC 1033

cb001F ------------------------------------------------------------ 902

cb001R GTAAAGTTAGATCAGATTGCAGACCAGTTAAGATCTATGCTGATGCTACTTTGGTTTTCC 842

BmDHS CTTTGTTGGTTGCTAAGACTTTCGCTAGACATGTCCAACAAAAACA-CTCCGAATTGCAA 1092

cb001F ------------------------------------------------------------ 902

cb001R CTTTGTTGGTTGCTAAGACTTTCGCTAGACATGTCCATTCAAAAACTCTCCGAATTGCAA 902

BmDHS GAAGCCTGA--------------------------------------------------- 1101

cb001F ------------------------------------------------------------ 902

cb001R GAAGCCTGACAGTAAAGGTGGATACGCATCCCTTCAGCTCCGTCGACAAGCTTGCGCCGC 962

BmDHS --------------------------------------------- 1101

cb001F --------------------------------------------- 902

cb001R ACTCGAGCACCACCACCACCACCACTGAGATTCCGGGCCTTAAAG 1007

**Multiple alignment of DNA sequencing– LmDHSp-cb001:**

CLUSTAL O(1.2.4) multiple sequence alignment

LmDHSp ------------------------------------------------------------ 0

cb001F CATTTTTGGTTTACTTTTAGAGAGATATACATATGCACCATCATCATCATCATTCTTCTG 60

cb001R ------------------------------------------------------------ 0

LmDHSp -------------------------------------ATGCTTGCCTCTG-CCCCAGCTC 22

cb001F GTGTAGATCTGGGAAGGTGGGCCTGTACTTCCAATCCATGCTTGCCTCTGCCCCAGCAAC 120

cb001R ------------------------------------------------------------ 0

LmDHSp CAAGGCCGGCCAAGAAGGACTCCGCTGCGTCCCGTAGGAAATCGGCGTCGAAGAGCACGG 82

cb001F CAAGGCCGGCCAAGAAGGACTCCGCTGCGTCCCGTAGGAAATCGGCGTCGAAGAGCACGG 180

cb001R ------------------------------------------------------------ 0

LmDHSp GAGCCGCTGTCAAGGATGACTCATCAGCGAGAGTTTCAGCGTCAGGTGCCGCTGAGTCGC 142

cb001F GAGCCGCTGTCAAGGATGACTCATCAGCGAGAGTTTCAGCGTCAGGTGCCGCTGAGTCGC 240

cb001R ------------------------------------------------------------ 0

LmDHSp CAGAGCAGAGCTGTACGCAGGTGCACGGCGTCGACTTCCAGTCGCTGGTGCACGCGACGC 202

cb001F CAGAGCAGAGCTGTACGCAGGTGCACGGCGTCGACTTCCAGTCGCTGGTGCACGCGACGC 300

cb001R --------------------------------------------------CTTGCATCAG 10

* ** *

LmDHSp AGGAGGAGACTCTCCGCGCTGTTGTGTCGAGCCTGCCCACGACTGGCCTGCAGGCTACCC 262

cb001F AGGAGGAGACTCTCCGCGCTGTTGTGTCGAGCCTGCCCACGACTGGCCTGCAGGCTACCC 360

cb001R GGGTGCTCATACCCCACAGAGTTGGGCCGTGGCGGGCGCC-------------------- 50

** * * * ** * **** * ** * * * * *

LmDHSp AGATTGGCCGTGCGCGCCAACTCGTGCAGCAGATTCTTCATCACCGCTCACCGGAGGATC 322

cb001F AGATTGGCCGTGCGCGCCAACTCGTGCAGCAGATTCTTCATCACCGCTCACCGGAGGATC 420

cb001R ---TACTCCGTGCAGGCAGATTGCTTACCATTCCACCGGCTCATCCGGAGAGGGATTCGA 107

* ****** ** * * * * *** * ***

LmDHSp GCGTCTTCCTCGCCTACACATCCA------ACATGATCTCATGTGGTCTTCGCGACACGT 376

cb001F GCGTCTTCCTCGCCTACACATCCA------ACATGATCTCATGTGGTCTTCGCGACACGT 474

cb001R GCGTCCTACTCGGCTACACATTCCAAACATGAATCTCCATGTTGGTCTTCGGCGACACGT 167

***** * **** ******** * ** * * * * *********

LmDHSp TTACGTACTTGGC--TCGCGAGCGGCTGGTGGACTGCTTCATCTCGTCCGCCGGCGGC-A 433

cb001F TTACGTACTTGGC--TCGCGAGCGGCTGGTGGACTGCTTCATCTCGTCCGCCGGCGGC-A 531

cb001R TTACGTACTTTGACTCGCGAGCGGGCTGGTGGACTGCTTCATCTCGTCCGCCGGCGGGCA 227

********** * ********************************** *

LmDHSp TCGAAGAGGACGTTAT-AAAGTGCGGCGGCAGCACGCTGCT-CGGCCAGTTCGGCCTGGA 491

cb001F TCGAAGAGGACGTTAT-AAAGTGCGGCGGCAGCACGCTGCT-CGGCCAGTTCGGCCTGGA 589

cb001R TCGAAGAGGACGTTATAAAAGTGCGGCGGCAGCACGCTGCTCGGCTCAGTTCGGCCTGGA 287

**************** ************************ * **************

LmDHSp CGGGCGAGCGCTGCGCCGCCGCGGCATCAACCGCATCGGCAACCTCCTCGTGCCCAACGA 551

cb001F CGGGCGAGCGCTGCGCCGCCGCGGCATCAACCGCATCGGCAACCTCCTCGTGCCCAACGA 649

cb001R CGGGCGAGCGCTGCGCCGCCGCGGCATCAACCGCATCGGCAACCTCCTCGTGCCCAACGA 347

************************************************************

LmDHSp CAACTACTGCTGGTTTG-AAGACTTCTTCACACCTGTGCTGGAGTCGGTTCAGGAGGCGC 610

cb001F CAACTACTGCTGGTTTG-AAGACTTCTTCACACCTGTGCTGGAGTCGGTTCAGGAGGCGC 708

cb001R CAACTACTGCTGGTTTGAAAGACTTCTTCACACCTGTGCTGGAGTCGGTTCAGGAGGCGC 407

***************** ******************************************

LmDHSp AGCGGGCGTCGCGATGGAAGACGCACACGGCCCCGTCCGAGTTCATTGAGGCCATGGGCG 670

cb001F AGCGGGCGTCGCGATGGAAGACGCACACGGCCCCGTCCGAGTTCATTGAGGCCATGGGCG 768

cb001R AGCGGGCGTCGCGATGGAAGACGCACACGGCCCCGTCCGAGTTCATTGAGGCCATGGGCG 467

************************************************************

LmDHSp CCGCCATCGCAAAGAACCACCCCGACACATGCACCTCCAGCCTGGTCTACTGGTGCTACC 730

cb001F CCGCCATCGCAAAGAACCACCCCGACACATGCACCTCCAGCCTGGTCTACTGGTGCTACC 828

cb001R CCGCCATCGCAAAGAACCACCCCGACACATGCACCTCCAGCCTGGTCTACTGGTGCTACC 527

************************************************************

LmDHSp GGAACGGGATCTCCGTCTTCTCGCCAGCTTTCACAGATGGCTCGATGGGAGACATGATTT 790

cb001F GGGAACGGATCTCCGTCTTCTCGCCAGCTTTTCACAGATGGCTTCGATGGGAGAACATGG 888

cb001R GGAACGGGATCTCCGTCTTCTCGCCAGCTTTCACAGATGGCTCGATGGGAGACATGATTT 587

** * ************************* * * ** * **

LmDHSp ACTTCTACAACTTCTCGCACAAGGGGCTCGTCGTGGACCCCCTGGAGGATGTTGTGCGGC 850

cb001F ------------------------------------------------------------ 888

cb001R ACTTCTACAACTTCTCGCACAAGGGGCTCGTCGTGGACCCCCTGGAGGATGTTGTGCGGC 647

LmDHSp TGCGCAAGCTGGCAGCAAAGGAGAAGGGCCGCAACTTGGCCATCGTGCTCGGCGGCGGTC 910

cb001F ------------------------------------------------------------ 888

cb001R TGCGCAAGCTGGCAGCAAAGGAGAAGGGCCGCAACTTGGCCATCGTGCTCGGCGGCGGTC 707

LmDHSp TTCCCAAGCACCACCTGCTGCGCAATGTGTCGATGGATGCTGTTGTCATGGTGACAACAG 970

cb001F ------------------------------------------------------------ 888

cb001R TTCCCAAGCACCACCTGCTGCGCAATGTGTCGATGGATGCTGTTGTCATGGTGACAACAG 767

LmDHSp GCTTGGAGGCCGATGGCTGCGTCAGCTCCGGCGTTCTCGCTGACGACGTGGCGTGCGGCC 1030

cb001F ------------------------------------------------------------ 888

cb001R GCTTGGAGGCCGATGGCTGCGTCAGCTCCGGCGTTCTCGCTGACGACGTGGCGTGCGGCC 827

LmDHSp TGCTCAGAGAGGAAACGGAGACTGTGCGCGTGCAAGGCGATGCCACGGTGGTATTCCCGC 1090

cb001F ------------------------------------------------------------ 888

cb001R TGCTCAGAGAGGAAACGGAGACTGTGCGCGTGCAAGGCGATGCCACGGTGGTATTCCCGC 887

LmDHSp TGATGCTGATTGCAGAGAAGGCTGCCACCCTGGAGGGGGCTGCAGCGTAA---------- 1140

cb001F ------------------------------------------------------------ 888

cb001R TGATGCTGATTGCAGAGAAGGCTGCCACCCTGGAGGGGGCTGCAGCGTGACAGTAAAGGT 947

LmDHSp ------------------------------------------------------------ 1140

cb001F ------------------------------------------------------------ 888

cb001R GGATACGGATCCGAATTCGAGCTCCGTCGACAAGCTTGCGGCCGCACTCGAGCACCACCA 1007

LmDHSp ----------------------------- 1140

cb001F ----------------------------- 888

cb001R CCACCACCATTGAGATCCCGGGTAAAAAG 1036

**Multiple alignment of DNA sequencing– LmDHSc-cb001:**

CLUSTAL O(1.2.4) multiple sequence alignment

LmDHSc ------------------------------------------------------------ 0

cb001F GCATTTTGGGGGTTACTTTTAGACGAGATATACATATGCACCATCATCATCATCATTCTT 60

cb001F2 ------------------------------------------------------------ 0

cb001R ------------------------------------------------------------ 0

LmDHSc ---------------------------------------ATGGCGAATATTGCGGAGTCT 21

cb001F CTGGTGTAGATCTGGGAAGCGGGCCTGTACTTCCAATCCATGGCGAATATTGCGGAGTCT 120

cb001F2 ------------------------------------------------------------ 0

cb001R ------------------------------------------------------------ 0

LmDHSc GCCGTGCTTGTGTCCTCGGCCTCCTCGGCGCAGGCGGTCGCGAAGCTGACACAAGTTCAG 81

cb001F GCCGTGCTTGTGTCCTCGGCCTCCTCGGCGCAGGCGGTCGCGAAGCTGACACAAGTTCAG 180

cb001F2 ------------------------------------------------------------ 0

cb001R ------------------------------------------------------------ 0

LmDHSc GGCCCCACGTCGGGCTTCGACAAGGCCCAACACATCATCGGTTCCTACTCAACAATGGGA 141

cb001F GGCCCCACGTCGGGCTTCGACAAGGCCCAACACATCATCGGTTCCTACTCAACAATGGGA 240

cb001F2 ------------------------------------------------------------ 0

cb001R ------------------------------------------------------------ 0

LmDHSc TTCCAGGCGACAAACTACGGTCTCGCCCGCTCCATCGCCCAGCGCATGATTCGAAAGCAG 201

cb001F TTCCAGGCGACAAACTACGGTCTCGCCCGCTCCATCGCCCAGCGCATGATTCGAAAGCAG 300

cb001F2 ------------------------------------------------------------ 0

cb001R ------------------------------------------------------------ 0

LmDHSc CCCCCTTCGAAGGTCTATCAAATAAAGGATGGCAAGTACGTACTGGTGCCGCCCGATGTC 261

cb001F CCCCCTTCGAAGGTCTATCAAATAAAGGATGGCAAGTACGTACTGGTGCCGCCCGATGTC 360

cb001F2 ------------------------------------------------------------ 0

cb001R ------------------------------------------------------------ 0

LmDHSc GGCGAGGACGGGAGGACGTTGCAACAAGAGCATGTCTACCCGAACCTGTTCATGGGTGTG 321

cb001F GGCGAGGACGGGAGGACGTTGCAACAAGAGCATGTCTACCCGAACCTGTTCATGGGTGTG 420

cb001F2 ------------------------------------------------------------ 0

cb001R ------------------------------------------------------------ 0

LmDHSc TCTGCCAACCTCATGGGCACTGGTTGTCGCGAGGCGGTTCGGTTTCTTGTGCAGGAGGGT 381

cb001F TCTGCCAACCTCATGGGCACTGGTTGTCGCGAGGCGGTTCGGTTTCTTGTGCAGGAGGGT 480

cb001F2 ------------------------------------------------------------ 0

cb001R ------------------------------------------------------------ 0

LmDHSc GTTGCCCATCGCTCGCCCGAAGCGTCTGCTGCCGCGTCAGCAGACGGAACAGACGACCAG 441

cb001F GTTGCCCATCGCTCGCCCGAAGCGTCTGCTGCCGCGTCAGCAGACGGAACAGACGACCAG 540

cb001F2 ------------------------------------------------------------ 0

cb001R ------------------------------------------------------------ 0

LmDHSc CTAATGTTTGCGCGACTCAAGAGGGAGTACGTCGAGACGTACGGTGGCCCTCCACACCCG 501

cb001F CTAATGTTTGCGCGACTCAAGAGGGAGTACGTCGAGACGTACGGTGGCCCTCCACACCCG 600

cb001F2 -----------------------AGAGGAGGTTTTGATT---TCATCATGAGACTCAACC 34

cb001R ------------------------------------------------------------ 0

LmDHSc GACGAGGAGGTACCTCGTGCTCACAGCTTCCTGTGCGCCATCGTCGTGAGCGGCGGTGGT 561

cb001F GACGAGGAGGTACCTCGTGCTCACAGCTTCCTGTGCGCCATCGTCGTGAGCGGCGGTGGT 660

cb001F2 CGGAGAGAGGTACCTCGTGCTCACAGCTTCCTGTGCGCCATCGTCGTGAGGG-AGGGGGT 93

cb001R ------------------------------------------------------------ 0

LmDHSc GTGGAGCACGACCTGCGCCGCGCCTGCACGGCTTACACACTGCACTACTACGCCAGCGAA 621

cb001F GTGGAGCACGACCTGCGCCGCGCCTGCACGGCTTACACACTGCACTACTACGCCAGCGAA 720

cb001F2 GTGGAGCACGACCTGCGCCGCGCCTGCACGGCTTACACACTGCACTACTACGCCAGCGAA 153

cb001R ------------------------------------------------------------ 0

LmDHSc GCACAAGGACATGTAAGCAGCACCATCTCCTCCGAGGCCACAGCGCCGTTGGAAGGCCTA 681

cb001F GCACAAGGACATGTAAGCAGCACCATCTCCTCCGAGGCCACAGCGCCGTTGGAAAGGCCT 780

cb001F2 GCACAAGGACATGTAAGCAGCACCATCTCCTCCGAGGCCACAGCGCCGTTGGAAGGCCTA 213

cb001R ------------------------------------------------------------ 0

LmDHSc CAGCAGCGGG--CGGAAACGCCTCTCGGCACTGGCGCGGCGGCCGGCGCAG-CGAAGCCA 738

cb001F ACAGCAGCGGGCGGGAAACGCCTCTCGGCACTGGCGCGGCGGGCCGGCGCAGCGAAGCCA 840

cb001F2 CAGCAGCGGG--CGGAAACGCCTCTCGGCACTGGCGCGGCGGCCGGCGCAG-CGAAGCCA 270

cb001R ------------------------------------------------------------ 0

LmDHSc -GCGCGCTTC-GGCAACGTCGAGTACCCACCGCAGGGCAGCCCCGGCTCTGCGCTATTCG 796

cb001F AGCGCGCTTCGGGCAACGTCGAGGTACCCCCACCCGCCAGGGGCAGGCCCCCGGGCTTCT 900

cb001F2 -GCGCGCTTC-GGCAACGTCGAGTACCCACCGCAGGGCAGCCCCGGCTCTGCGCTATTCG 328

cb001R ------------------------------------------------------------ 0

LmDHSc ACCGCCTCATGCGCACATTTGCTCAGCGCCTCTGCGCCCGCCAGGCACGGTTGCGTGCGG 856

cb001F TTG--------------------------------------------------------- 903

cb001F2 ACCGCCTCATGCGCACATTTGCTCAGCGCCTCTGCGCCCGCCAGGCACGGTTGCGTGCGG 388

cb001R ------------------------------------------------------------ 0

LmDHSc CTGCTATGGCGAAGCCGATTCCTGACAAGTACGACGACGTTTGTAGCTGGTCCGTCACTC 916

cb001F ------------------------------------------------------------ 903

cb001F2 CTGCTATGGCGAAGCCGATTCCTGACAAGTACGACGACGTTTGTAGCTGGTCCGTCTCTC 448

cb001R ------------------------------------------------------------ 0

LmDHSc CCAGCGAGGTGTGGGCGCTGTGCGGGTTGTGGCTGGTCGACATGCTCGCGGAGGCGCTGA 976

cb001F ------------------------------------------------------------ 903

cb001F2 TTTGCGAGGTGTGGGCGCTGTGCGGGTTGTGGCTGGTCGACATGCTCGCGGAGGCGCTGA 508

cb001R ------------------------------------------------------------ 0

LmDHSc GGGCTGTGCAGAGCTGTCCGTCTCACTTGACGAGCGGTTCTGGGGTGGGCACTGCAGAAT 1036

cb001F ------------------------------------------------------------ 903

cb001F2 GGGCTGTGCAGAGCTGTCCGTCTCACTTGACGAGCGGTTCTGGGGTGGGCACTGCAGAAT 568

cb001R ------------------------------------------------------------ 0

LmDHSc CCGTCACCGCGAACGGGAAGGGGCAGGAAGCAGATCGAGATGCACACATTGCTACATCGG 1096

cb001F ------------------------------------------------------------ 903

cb001F2 CCGTCACCGCGAACGGGAAGGGGCAGGAAGCAGATCGAGATGCACACATTGCTACATCGG 628

cb001R ------------------------------------------------------------ 0

LmDHSc CGTTGTATCGTGCGGAAGCACTCGCCAGGGCACGCACAACTGTCGTCTACTGGGCAGCCG 1156

cb001F ------------------------------------------------------------ 903

cb001F2 CGTTGTATCGTGCGGAAGCACTCGCCAG-GCACGCACAACTGTCGTCTACTGGGCAGCCG 687

cb001R ---------------TTCGGCAAGGGGCAACGCACAAACTGTCGTCTACTGGGGCAGCCG 45

LmDHSc TGCAGCAGGTCTCTCTGTTCAGCCCTTCCTTCGTC-GACGGCGACATCACGAGCTACCTG 1215

cb001F ------------------------------------------------------------ 903

cb001F2 TGCAGCAGGTCTCTCTGTTCAGCCCTTCCTTCGTC-GACGGCGACATCACGAGCTACCTG 746

cb001R TGCAGCAGGTCTCTCTGTTCAGCCCTTCCATCGTCGAACGGCGACATCACGAGCTACCTG 105

LmDHSc TTGCCCACGCCCGTCCCTGCAGCCCGTCCTGCGCACCGAAAAGGCGGTTC--AGTT-GCG 1272

cb001F ------------------------------------------------------------ 903

cb001F2 TTGCCCACGCCGTTCCCTGCAGCCCGTTCTGCGCACCGAAAAAGGGCGGTTCAGTTGCGG 806

cb001R TTGCCCACGCCCGTCCCTGCAGCCCGTCCTGCGCACCGAAAAAGGCGGTTC--AGTTGCG 163

LmDHSc GACGAGAGCGCTGCCAATTCGAAGGAGTTGAAGAG--ACGTCGAAAAGCCTCCTCGTCTT 1330

cb001F ------------------------------------------------------------ 903

cb001F2 AACGAGAGCGCTGCCAATTCGAACGAAGTTTGAAGAGACGTCGAAAAAGGCCTTCCTTCG 866

cb001R GACGAGAGCGCTGCCAATTCGAAGGAGTTGAAGAG--ACGTCGAAAAGCCTCCTCGTCTT 221

LmDHSc CGC------CCACGGCGGCCACCGCGGTGGAG--GACGAACCTCCGGTGGTGGAGCGCCT 1382

cb001F ------------------------------------------------------------ 903

cb001F2 GTCTTCGGCCCACGCCGGTCAACCCGTCGGTGGGAAGGAACGAAACCTTC---------- 916

cb001R CGC------CCACGGCGGCC--ACCGCGGTGGAGGACGAACCTCCGGTGGTGGAGCGCCT 273

LmDHSc GCAGATCGATCTCGTCCGTGACGTGTACTCGATCAACAAGCTCGCCATGCTCAGCAAGAA 1442

cb001F ------------------------------------------------------------ 903

cb001F2 CCCGGTGGATGTAGAG-------------------------------------------- 932

cb001R GCAGATCGATCTCGTCCGTGACGTGTACTCGATCAACAAGCTCGCCATGCTCAGCAAGAA 333

LmDHSc GACCGGCATGCTCATCTGTGGTGGTGGTGTCGTGAAGCATCACGTATGTAACGCCAACCT 1502

cb001F ------------------------------------------------------------ 903

cb001F2 ------------------------------------------------------------ 932

cb001R GACCGGCATGCTCATCTGTGGTGGTGGTGTCGTGAAGCATCACGTATGTAACGCCAACCT 393

LmDHSc CATGCGCAACGGCGCGGACTTCACCATCATCCTGAACAACGGGCAGGAGTTCGACGGCTC 1562

cb001F ------------------------------------------------------------ 903

cb001F2 ------------------------------------------------------------ 932

cb001R CATGCGCAACGGCGCGGACTTCACCATCATCCTGAACAACGGGCAGGAGTTCGACGGCTC 453

LmDHSc TGACGCCGGTGCCAAACCCGAGGAGGCACTTTCGTGGGGGAAGGTACGGATGGAAGGGGC 1622

cb001F ------------------------------------------------------------ 903

cb001F2 ------------------------------------------------------------ 932

cb001R TGACGCCGGTGCCAAACCCGAGGAGGCACTTTCGTGGGGGAAGGTACGGATGGAAGGGGC 513

LmDHSc GTTCGTGAAGGTGTATGGAGAGGTCAGCACGTACCTCCCGCTCCTGGTGGCGGACGTTTT 1682

cb001F ------------------------------------------------------------ 903

cb001F2 ------------------------------------------------------------ 932

cb001R GTTCGTGAAGGTGTATGGAGAGGTCAGCACGTACCTCCCGCTCCTGGTGGCGGACGTTTT 573

LmDHSc CGTGCCGGCGGTGCGGCAGCGTAGGGCTACGGACGACGCGCAGCCGCGCAGGAGGCAATC 1742

cb001F ------------------------------------------------------------ 903

cb001F2 ------------------------------------------------------------ 932

cb001R CGTGCCGGCGGTGCGGCAGCGTAGGGCTACGGACGACGCGCAGCCGCGCAGGAGGCAATC 633

LmDHSc CTCGCGAGGCGCGCGTCTGCCGCAAGACGTCTCCGGGCACTCTCACCTGTGTCGTGGAGA 1802

cb001F ------------------------------------------------------------ 903

cb001F2 ------------------------------------------------------------ 932

cb001R CTCGCGAGGCGCGCGTCTGCCGCAAGACGTCTCCGGGCACTCTCACCTGTGTCGTGGAGA 693

LmDHSc ATGA-------------------------------------------------------- 1806

cb001F ------------------------------------------------------------ 903

cb001F2 ------------------------------------------------------------ 932

cb001R ATGACAGTAAAGGTGGATACGGATCCGAATTCGAGCTCCGTCGACAAGCTTGCGGCCGCA 753

LmDHSc ------------------------------------------ 1806

cb001F ------------------------------------------ 903

cb001F2 ------------------------------------------ 932

cb001R CTCGAGCACCACCACCACCACCAATAGATCCCGGGTAAAAAC 795
