# Supplementary material for: Structural features and development of an assay platform of the parasite target deoxyhypusine synthase of Brugia malayi and Leishmania major
Source: PLoS Negl Trop Dis. 2020 Oct 12;14(10):e0008762. doi: 10.1371/journal.pntd.0008762 (PMC7581365; doi:10.1371/journal.pntd.0008762)
Supplement: S1 Table — (DOCX) [file pntd.0008762.s014.docx]

**S1 Table. Oligonucleotide sequences.**

| Oligonucleotide | Sequence 5’ to 3’ |
| --- | --- |
| Bm F1 | **TACTTCCAATCC**ATGGACAACGGTAACTGTAAATTCGATGTTC |
| Bm F2 | **TACTTCCAATCCATG**GGTAACTGTAAATTCGATGTTCATATCGCC |
| Bm F3 | **TACTTCCAATCCATG**AAATTCGATGTTCATATCGCCGAAATGT |
| Bm F4 | **TACTTCCAATCCATG**GATGTTCATATCGCCGAAATGTCC |
| Bm R1 | **TATCCACCTTTACTGTCA**GGCTTCTTGCAATTCGGA |
| Lmc F1 | **TACTTCCAATCC**ATGGCGAATATTGCGGAGTCTGC |
| Lmc R1 | **TATCCACCTTTACTGTCA**TTCTCCACGACACAGGTG |
| Lmc R2 | **TATCCACCTTTACTGTCA**GGAGACGTCTTGCGGCAG |
| Lmc R3 | **TATCCACCTTTACTGTCA**CGAGGATTGCCTCCTGCG |
| Lmc R4 | **TATCCACCTTTACTGTCA**CGGCTGCGCGTCGTCCGT |
| Lmp F1 | **TACTTCCAATCC**ATGCTTGCCTCTGCCCCAGC |
| Lmp F2 | **TACTTCCAATCCATG**CCGGCCAAGAAGGACTCC |
| Lmp F3 | **TACTTCCAATCCATG**TCCGCTGCGTCCCGTAGG |
| Lmp F4 | **TACTTCCAATCCATG**AAGGATGACTCATCAGCGAGAGT |
| Lmp R1 | **TATCCACCTTTACTGTCA**CGCTGCAGCCCCCTCCAG |

Bold nucleotides denote ligation independent cloning sites.
