# Supplementary material for: Structural features and development of an assay platform of the parasite target deoxyhypusine synthase of Brugia malayi and Leishmania major
Source: PLoS Negl Trop Dis. 2020 Oct 12;14(10):e0008762. doi: 10.1371/journal.pntd.0008762 (PMC7581365; doi:10.1371/journal.pntd.0008762)
Supplement: S2 Table — (DOCX) [file pntd.0008762.s015.docx]

**S2 Table. Detailed information for BmDHS and LmDHS constructs.**

| Construct ID | Primer combination | | Amplicon size (bp) | Molecular weight (Da) | |
| --- | --- | --- | --- | --- | --- |
|  | **Forward^a^** | **Reverse^a^** |  | **with tag** | **no tag** |
| BmDHS-cb001 | Bm F1 (1) | Bm R1 (366) | 1128 | 43,548.6 41,083.0 | |
| BmDHS-cb002 | Bm F2 (4) | Bm R1 (366) | 1122 | 43,319.4 40,853.8 | |
| BmDHS-cb003 | Bm F3 (7) | Bm R1 (366) | 1113 | 43,045.1 40,579.5 | |
| BmDHS-cb004 | Bm F4 (9) | Bm R1 (366) | 1107 | 42,769.7 40,304.1 | |
| LmDHSc-cb001 | Lmc F1 (1) | Lmc R1 (601) | 1833 | 66,890.7 64,425.1 | |
| LmDHSc-cb002 | Lmc F1 (1) | Lmc R4 (576) | 1758 | 64,062.6 61,597.0 | |
| LmDHSc-cb003 | Lmc F1 (1) | Lmc R3 (582) | 1776 | 64,833.4 62,367.8 | |
| LmDHSc-cb004 | Lmc F1 (1) | Lmc R2 (592) | 1806 | 65,913.6 63,448.0 | |
| LmDHSp-cb001 | Lmp F1 (1) | Lmp R1 (379) | 1167 | 43,377.7 40,912.0 | |
| LmDHSp-cb002 | Lmp F2 (10) | Lmp R1 (379) | 1143 | 42,613.8 40,148.2 | |
| LmDHSp-cb003 | Lmp F3 (15) | Lmp R1 (379) | 1128 | 42,074.2 39,608.5 | |
| LmDHSp-cb004 | Lmp F4 (32) | Lmp R1 (379) | 1077 | 40,457.4 37,991.8 | |

^a^ Numbers in parentheses indicate first and last amino acid according to predicted gene open reading frame.
