# Supplementary material for: Structural features and development of an assay platform of the parasite target deoxyhypusine synthase of Brugia malayi and Leishmania major
Source: PLoS Negl Trop Dis. 2020 Oct 12;14(10):e0008762. doi: 10.1371/journal.pntd.0008762 (PMC7581365; doi:10.1371/journal.pntd.0008762)
Supplement: S3 Table — (DOCX) [file pntd.0008762.s016.docx]

**S3 Table.**  **Comparison of active site amino acids between BmDHS and its counterparts in *H. sapiens* (HsDHS), *T. brucei* (TbDHSp/DHSc) and *L. major* (LmDHSp/DHSc).**

| **HsDHS ^a^** | **BmDHS ^a^** | **Dead site ^a^** | | **Catalytic site ^a^** | |  |
| --- | --- | --- | --- | --- | --- | --- |
|  |  | **TbDHSp/**  **DHSc** | **LmDHSp/**  **DHSc ^b^** | **TbDHSp/**  **DHSc** | **LmDHSp/**  **DHSc ^b^** |  |
| **NAD^+^ 4-Å shell** | | | | | | |
| F54(A1) | F55(A1) | L40p(A1) | Q47p(A1) | F49c(A2) | F48c(A2) |  |
| T104(A1) | T101(A1) | T98c(A2) | S108c(A2) | T71p(A1) | T114p(A1) |  |
| S105(A1) | S102(A1) | A99c(A2) | A109c(A2) | S72p(A1) | S115p(A1) |  |
| N106(A1) | N103(A1) | N100c(A2) | N110c(A2) | N73p(A1) | N116p(A1) |  |
| L107(A1) | L104(A1) | L101c(A2) | L111c(A2) | L74p(A1) | M117p(A1) |  |
| S109(A1) | T106(A1) | G103c(A2) | G113c(A2) | S76p(A1) | S119p(A1) |  |
| T131(A1) | S128(A1) | S161c(A2) | S184c(A2) | T98p(A1) | S141(A1) |  |
| A132(A1) | A129(A1) | G162c(A2) | G185c(A2) | A99p(A1) | A142p(A1) |  |
| G133(A1) | G130(A1) | G163c(A2) | G186c(A2) | G100p(A1) | G143p(A1) |  |
| E136(A1) | E133(A1) | E166c(A2) | E189c(A2) | E103p(A1) | E146p(A1) |  |
| E137(A1) | E134(A1) | H167c(A2) | H190c(A2) | E104p(A1) | E147p(A1) |  |
| A235(A1) | A232(A1) | S323c(A2) | S295c(A2) | A210p(A1) | A253p(A1) |  |
| D238(A1) | D235(A1) | D326c(A2) | D398c(A2) | D213p(A1) | D256p(A1) |  |
| G239(A1) | G236(A1) | G327c(A2) | G399c(A2) | G214p(A1) | G257p(A1) |  |
| S240(A1) | S237(A1) | D328c(A2) | D400c(A2) | S215p(A1) | S258p(A1) |  |
| G282(A1) | G279(A1) | G371c(A2) | G488c(A2) | G260p(A1) | G301p(A1) |  |
| G283(A1) | G280(A1) | G372c(A2) | G489c(A2) | A261p(A1) | G302p(A1) |  |
| I306(A1) | V303(A1) | L395c(A2) | L512c(A2) | V280p(A1) | V321p(A1) |  |
| N307(A1) | N304(A1) | N396c(A2) | N513c(A2) | T281p(A1) | T322p(A1) |  |
| T308(A1) | T305(A1) | N397c(A2) | N514c(A2) | T282p(A1) | T323p(A1) |  |
| A309(A1) | G306(A1) | A398c(A2) | G515c(A2) | G283p(A1) | G324p(A1) |  |
| S317(A1) | S314(A1) | A406c(A2) | A523c(A2) | S291p(A1) | S332p(A1) |  |
| A341(A1) | A338(A1) | S430c(A2) | G547c(A2) | G315p(A1) | G356p(A1) |  |
| D342(A1) | D339(A1) | E431c(A2) | E548c(A2) | D316p(A1) | D357p(A1) |  |
| A343(A1) | A340(A1) | V432c(A2) | V549c(A2) | A317p(A1) | A358p(A1) |  |
| G284(A2) | G281(A2) | G262p(A1) | G303p(A1) | G373c(A2) | G490c(A2) |  |
| V285(A2) | V282(A2) | L263p(A1) | L304p(A1) | V374c(A2) | V491c(A2) |  |
| H288(A2) | H285(A2) | H266(A1) | H307p(A1) | H377c(A2) | H494c(A2) |  |
| D313(A2) | D310(A2) | D287p(A1) | D328p(A1) | D402c(A2) | D519c(A2) |  |
| S315(A2) | S312(A2) | C289p(A1) | C330p(A1) | S404c(A2) | S521c(A2) |  |
| D316(A2) | D313(A2) | E290p(A1) | V331p(A1) | D405c(A2) | D522c(A2) |  |
| S317(A2) | S314(A2) | S291p(A1) | S332p(A1) | A406c(A2) | A523c(A2) |  |
| **GC7 4-Å shell** | | | | | | |
| N106(A1) | N103(A1) | N100c(A2) | N110c(A2) | N73p(A1) | N116p(A1) |  |
| R165(A1) | R162(A1) | H207c(A2) | R248c(A2) | R132p(A1) | R175p(A1) |  |
| I166(A1) | A163(A1) | F208c(A2) | F249c(A2) | V133p(A1) | I176p(A1) |  |
| G167(A1) | G164(A1) | G209c(A2) | G250c(A2) | G134p(A1) | G177p(A1) |  |
| V171(A1) | I168(A1) | Y213c(A2) | Y254c(A2) | V138p(A1) | V181p(A1) |  |
| S240(A1) | S237(A1) | D328c(A2) | D400c(A2) | S215p(A1) | S258p(A1) |  |
| D243(A1) | D240(A1) | E331c(A2) | S403c(A2) | D218p(A1) | D261p(A1) |  |
| H288(A2) | H285(A2) | H266p(A1) | H307p(A1) | H377c(A2) | H494c(A2) |  |
| N292(A2)’ | N289(A2) | R270p(A1) | R311p(A1) | N381c(A2) | N498c(A2) |  |
| L295(A2) | L292(A2) | - | - | L384c(A2) | L501c(A2) |  |
| G314(A2) | G311(A2) | G288p(A1) | G329p(A1) | G403c(A2) | G520c(A2) |  |
| S315(A2) | S312(A2) | C289p(A1) | C330p(A1) | S404c(A2) | S521c(A2) |  |
| D316(A2) | D313(A2) | E290p(A1) | V331p(A1) | D405c(A2) | D522c(A2) |  |
| E323(A2) | E320(A2) | A297p(A1) | D338p(A1) | E412c(A2) | E529c(A2) |  |
| W327(A2) | W324(A2) | N301p(A1) | C342p(A1) | W416c(A2) | W533c(A2) |  |
| K329*(A2) | K326*(A2) | L303p(A1) | L344p(A1) | K418c*(A2) | K535c*(A2) |  |

**^a^** - analysis for the active site formed by protomers A1 and A2.

**^b^** - active-site residues for LmDHS paralogs based on primary sequence alignment and homology model.

A1- residue from protomer A1 (see text).

A2- residue from protomer A2 (see text).

c: residue from catalytically-active protomer from LmDHS and TbDHS.

p: residue from catalytically-dead protomer from LmDHS and TbDHS.

* catalytic lysine residue
